# Integrated immune, hormonal, and transcriptomic profiling reveals sex-specific dysregulation in long COVID patients with ME/CFS

**DOI:** 10.1016/j.xcrm.2025.102449

**Published:** 2025-11-07

**Authors:** Shima Shahbaz, Mohammed Osman, Hussain Syed, Andrew Mason, Rhonda J. Rosychuk, Jan Willem Cohen Tervaert, Shokrollah Elahi

**Affiliations:** 1 Mike Petryk School of Dentistry, Division of Foundational Sciences, University of Alberta, Edmonton, AB T6G 2E1, Canada; 2 Department of Medicine, Division of Rheumatology, University of Alberta, Edmonton, AB T6G 2E1, Canada; 3 Li Ka Shing Institute of Virology, University of Alberta, Edmonton, AB T6G 2E1, Canada; 4 Women and Children Health Research Institute, University of Alberta, Edmonton, AB T6G 2E1, Canada; 5 Department of Medicine, Division of Gastroenterology, University of Alberta, Edmonton, AB T6G 2E1, Canada; 6 Department of Pediatrics, Division of Infectious Disease, University of Alberta, Edmonton, AB T6G 2E1, Canada; 7 Cancer Research Institute of Northern Alberta, University of Alberta, Edmonton, AB T6G 2E1, Canada; 8 Glycomics Institute of Alberta, University of Alberta, Edmonton, AB T6G 2E1, Canada; 9 Alberta Transplant Institute, Faculty of Medicine and Dentistry, University of Alberta, Edmonton, AB T6G 2E1, Canada

**Keywords:** Reelin, testosterone, chronic fatigue syndrome, sex-related differences, long COVID, galectin-9, cortisol, growth hormone, artemin, erythropoiesis

## Abstract

Long COVID (LC) manifests with sex-specific differences, particularly in those with myalgic encephalomyelitis/chronic fatigue syndrome (ME/CFS). Our study reveals that female LC patients (LCF) with ME/CFS show a shift toward myelopoiesis, reduced lymphocytes, increased neutrophils/monocytes, and depleted regulatory T cells—suggesting persistent immune activation. Elevated CD71^+^ erythroid cells and disrupted erythropoiesis contribute to fatigue and tissue damage in LCF. Cytokine profiling indicates a stronger pro-inflammatory response in LCF compared to males (LCM), along with markers of gut barrier dysfunction. Hormonal analysis shows reduced testosterone in LCF and estradiol in LCM. Transcriptomic data reveal neuroinflammatory signatures in LCF, potentially explaining cognitive symptoms. We also identify biomarkers that distinguish LCF from LCM and correlate with sex-specific clinical symptoms. Overall, LC with ME/CFS is characterized by sex-specific immune, hormonal, and transcriptional alterations, with females exhibiting more severe inflammation. These insights underscore the need for sex-tailored interventions, including consideration of hormone replacement therapy.

## Highlights

• Female long COVID (LC) patients show heightened immune activation and inflammation
• Disrupted sex hormone levels differ between male and female LC patients
• Neuroinflammatory gene signatures may explain cognitive symptoms in females
• Sex-specific biomarkers suggest need for tailored LC therapies

## Graphical abstract

**Figure:**
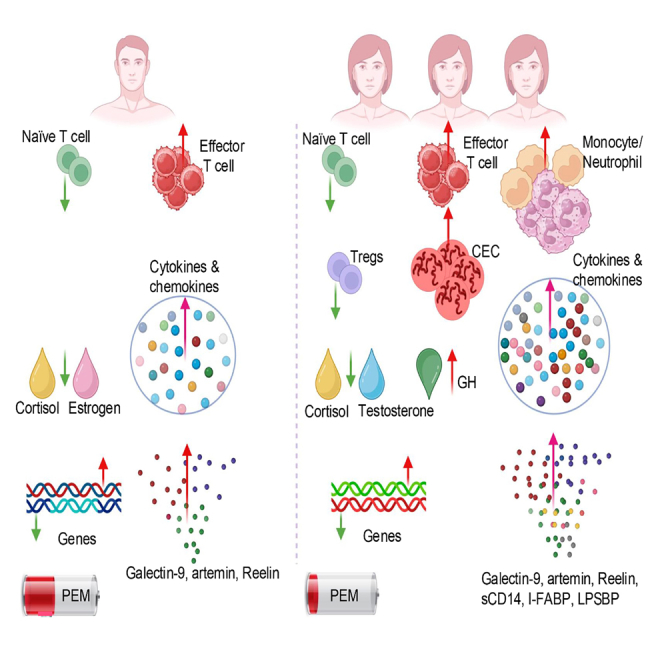

## Teaser

Shahbaz et al. find that female long COVID patients with ME/CFS exhibit heightened inflammation, altered hematopoiesis, disrupted hormone levels, and neuroinflammatory gene signatures. In parallel, our study identifies sex-specific immune and hormonal biomarkers that correlate with clinical symptoms, supporting the need for tailored therapeutic strategies, including consideration of hormone replacement therapy.

## Introduction

Since the onset of the SARS-CoV-2 pandemic, long COVID (LC), a heterogeneous condition marked by prolonged symptoms following acute infection, has become a major global health concern. Now in its fifth year, LC affects multiple organ systems and significantly impairs quality of life, often resulting in disability and inability to work. Symptoms vary in severity and may resemble those seen in myalgic encephalomyelitis/chronic fatigue syndrome (ME/CFS)^1^^,^^2^^,^^3^ or systemic autoimmune rheumatic diseases (SARDs).^4^

Any acute infectious disease that can damage multiple organs (e.g., cardiac, pulmonary, and/or renal involvement), such as COVID-19, can be associated with long-term sequelae. In some individuals with persistent, debilitating fatigue following SRAS-CoV-2 infection, documented damage to vital organs may explain their fatigue. However, many patients with LC develop severe fatigue after the acute infection despite having only mild symptoms and no discernible organ damage.

Several hypotheses have been proposed to explain LC, including immune dysregulation, impaired hematopoiesis, neuroendocrine abnormalities, genetic predisposition, compromised gastrointestinal integrity, and the presence of SARS-CoV-2 antigens in tissues.^5^^,^^6^^,^^7^^,^^8^ Persistent severe fatigue that occurs after an acute infection, in the absence of chronic cardiac, pulmonary, or renal dysfunction, is likely due to a state of chronic low-grade neuroinflammation.^9^ SARS-CoV-2 may persist or cause viral-associated damage to the brain,^10^^,^^11^ intestine, and liver, which can result in ongoing damage.^12^ It is known that SARS-CoV-2 enters the olfactory mucosa and can penetrate into the brain from the cribriform plate or via vagal pathways.^13^ Alternatively, the virus may directly translocate across the blood-brain barrier (BBB) due to increased permeability from inflammatory cytokines or cells (e.g., monocytes).^14^ Also, SARS-CoV-2 can reach neural tissue via circumventricular organs (CVOs).^15^ Hence, long-term neuropsychiatric symptoms may stem from chronic neuroinflammation and hypoxic injury.^15^ In line with these observations, our recent findings point to elevated levels of Galectin-9 (Gal-9), artemin (ARTN), and Reelin in the plasma of LC patients with ME/CFS.^8^^,^^16^^,^^17^ Gal-9 has immunomodulatory properties^18^ and is linked to cognitive impairments in people living with HIV.^19^^,^^20^ ARTN, a neurotrophic factor, influences sensory neuron function and osteoarthritis pain perception^21^^,^^22^ and correlates with pain indexes and cognitive impairment in LC patients with ME/CFS.^8^ Similarly, Reelin, a glycoprotein involved in neuronal migration and synaptic plasticity, has also been implicated in immune dysregulation and chronic inflammatory conditions.^23^ Importantly, ARTN is secreted by CD71^+^ erythroid cells (CECs),^8^^,^^24^ which expand during stress hematopoiesis^25^^,^^26^^,^^27^ and have been observed in the peripheral blood both during acute SARS-CoV-2 infection^28^^,^^29^ and in LC patients with ME/CFS.^8^ These cells may contribute to immune suppression through reactive oxygen species (ROS), arginase I/II activity, and cell-cell interactions^26^^,^^27^ and may promote fatigue and brain fog through the secretion of ARTN, providing a potential mechanistic link.

A striking observation in our cohort is the disproportionate representation of females (∼70%) among LC patients, a trend that has been reported in multiple studies.^8^^,^^17^^,^^30^ While sex differences in acute SARS-CoV-2 immune responses are well documented,^31^ less is known about their role in LC. Therefore, our study explores sex-specific immune, hormonal, and transcriptomic differences in LC patients with ME/CFS, with particular emphasis on immune cell phenotypes (e.g., increased neutrophils/monocytes and reduced lymphocytes in females), imbalanced sex hormone levels, and the potential role of CECs in contributing to fatigue. These findings may help explain why females are more susceptible to LC and offer insights into biological pathways that could be targeted in future interventions.

By investigating the interplay between immune dysregulation, hormonal imbalance, and erythropoietic dysfunction through a sex-specific lens, our study aims to advance understanding of LC/ME/CFS pathogenesis and identify key mechanisms underlying differential outcomes between males and females. In line with this aim, we observed distinct sex hormone imbalances in our cohort: females exhibited lower testosterone, males had reduced estradiol, and both sexes showed decreased cortisol levels. These findings suggest that personalized, sex-hormone-based therapies may hold promise as potential interventions for LC patients with ME/CFS.

We performed sex-based analyses in 78 LC individuals with ME/CFS and compared them with 62 age- and sex-matched recovered (R) individuals. These R individuals were infected with SARS-CoV-2 and did not develop complications related to LC more than 12 months after the acute infection. To better characterize these differences associated with sex and immunological signatures, we utilized a multipronged approach comprised of immunophenotyping, multiplex ELISAs, and whole blood bulk RNA sequencing (RNA-seq).

## Results

### Study cohorts

**Figure 1:**
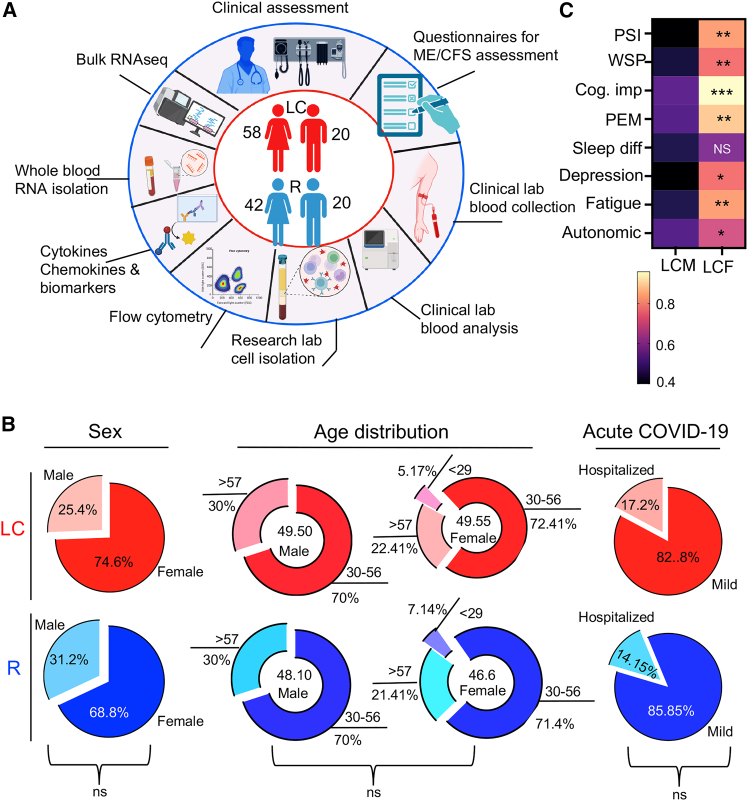
Study design, demographic, and clinical analysis of cohorts (A) Schematic of the study design. Numbers in center of diagram indicate participants in each study cohort (LC, 58 females and 20 males) (R, 42 females and 20 males). Outer ring indicates different assays performed on patients/samples. (B) Demographic characteristics for LC (top row, red) and R (bottom row, blue) displayed as ring charts. Center values in “age” are median ages in years. Acute COVID-19 disease severity indicates percentages of patients with mild symptoms or required hospitalization in LC vs. R. (C) Heatmap of symptoms colored according to symptom severity score, such as pain severity index (PSI), widespread pain index (WSP), cognitive impairment (Cog. imp; brain fog), post-exertional malaise (PEM), and sleep difficulty (sleep diff). LCM, long COVID male; LCF, long COVID female. *p* values were calculated using two tailed, Mann-Whitney t test ∗*p* < 0.05, ∗∗*p* <0.01, ∗∗∗*p* <0.001. ns, not significant.

Our cohort comprised 78 LC individuals with ME/CFS (58 females and 20 males), as well as 62 R individuals (42 females and 20 males) (Figure 1A and Table S1). Our demographic factors indicated that the LC and R groups were well matched in sex (74.4% female LCF vs. 67.7% female RF; 25.6% male LCM vs. 32.3% male RM) and age (female mean 49.5 ± 10.74 LC vs. 49.5 ± 14.6 male LC; female mean 45.6 ± 12.85 R vs. 48.25 ± 9.8 male R) (Figure 1B). Although we tried to match LC and R for age and sex, *in our LC cohort, females comprised 74.4% of participants, corresponding to an observed female-to-male ratio of 2.9:1* (Figure 1B). Analysis of elapsed days since the onset of acute SARS-CoV-2 infection revealed no significant difference between LC and R in our cohorts, as reported before.^8^ Notably, clinical scores of reported symptoms showed significantly higher symptoms burden and intensity in LCF compared to LCM, except for sleeping difficulties (Figure 1C).

### Differential immune cell phenotype in male and female LC patients

**Figure 2:**
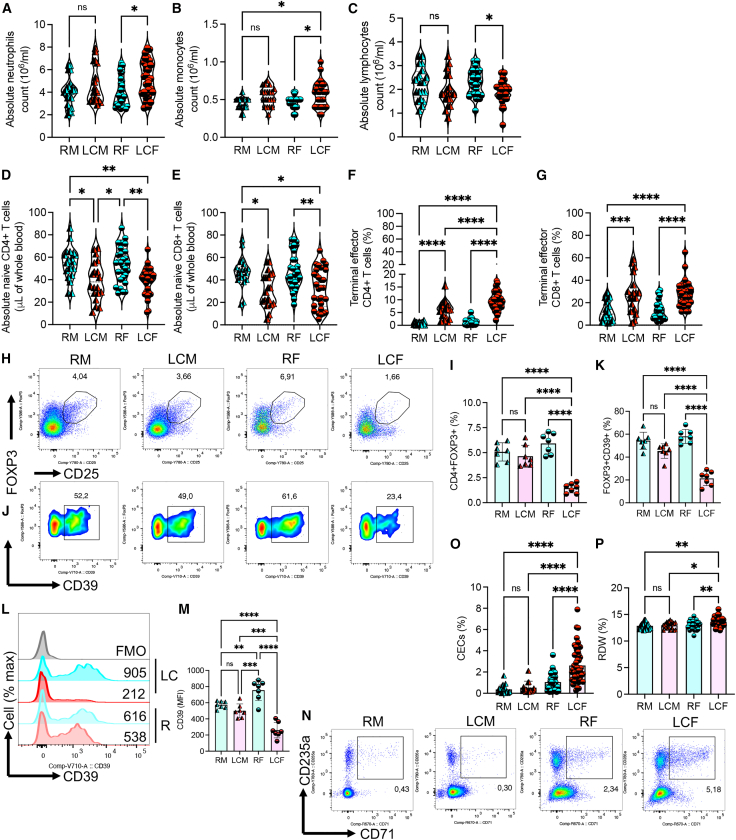
Sex-associated altered innate and adaptive immune cell phenotype (A–E) (A) Absolute number of neutrophils; (B) monocytes; (C) lymphocytes; (D) naive CD4^+^ T cell; (E) naive CD8^+^ T cell counts in the whole blood of RM, LCM, RF, and LCF. (F and G) (F) Cumulative data of terminal effector (TE) CD4^+^ and (G) terminal effector (TE) CD8^+^ T cells in peripheral blood mononuclear cells (PBMCs) of RM, LCM, RF, and LCF. (H and I) (H) Representative flow cytometry plots and (I) cumulative data of percentages of Tregs in PBMCs. (J and K) (J) Representative flow cytometry plots and (K) cumulative data of percentages of CD39^+^ cells among FOXP3^+^CD25^+^ cells in PBMCs. (L and M) (L) Representative flow cytometry histogram plots and (M) cumulative data of the intensity of CD39 expression measured by the mean fluorescence intensity (MFI) among Tregs in PBMCs. (N and O) (N) Representative flow cytometry plots and (O) cumulative data of percentages of CECs in PBMCs. (P) The percentages of RDW in the whole blood of different groups. *p* values were calculated using Kruskal-Wallis analysis with Dunn’s multiple comparisons test (A–G, I, K, M, O, P). The blue symbol represents R and red represents LC. The circle and triangle symbols represent male and female, respectively. ns, not significant. Error bars represent mean ± SD. ∗*p* < 0.05, ∗∗*p* <0.01, ∗∗∗*p* <0.001, ∗∗∗∗*p* <0.0001.

Compared to the RF group, the LCF shows a relative increase in absolute neutrophils and monocytes along with a decrease in lymphocytes in the complete blood cell count (Figures 2A–2C). However, this was not the case for the LCM vs. RM groups (Figures 2A–2C). Considering the reduction in lymphocyte count in LCF, we subjected the T cells to further analysis. Compared to the R groups, we found a significant reduction in the absolute number of naive (N) CD4^+^ and CD8^+^ T cells in the whole blood of both LCF and LCM cohorts (Figures 2D and 2E), with no significant changes in the absolute number of central memory (CM) and effector memory (EM) T cells, as previously reported.^8^ Although absolute counts of terminal effector (TE) T cells in whole blood were not assessed, we found a significant increase in the frequency of TE CD4^+^ and CD8^+^ T cell subsets (CD45RA^+^CCR7^+^CD95^+^CD28^−^)^8^ in peripheral blood mononuclear cells (PBMCs) from both male and female LC patients compared to R groups (Figures 2F, 2G, and S1A). Notably, we observed a significant reduction in the proportion of regulatory T cells (Tregs) in PBMCs of LCF compared to other groups (Figures 2H, 2I, and S1A). Additionally, the frequency of CD39-expressing Tregs and the intensity of CD39 were significantly reduced in LCF compared to other groups (Figures 2J–2M). Finally, we found a significant expansion of CECs in the peripheral blood of LCF compared to RF individuals (Figures 2N and 2O). In agreement with the observed unusual morphological abnormalities of red blood cells (RBCs) in acute COVID-19 patients,^32^ we noted a significant increase in the RBC distribution width (RDW), associated with mortality risk in COVID-19 patients,^32^ in the whole blood of LCF compared to other groups (Figure 2P). These findings suggest dysregulated hematopoiesis skews toward myelopoiesis at the expense of lymphopoiesis in LCF patients. Additionally, the reduction in naive T cells and the expansion of TE T cells support persistent T cell activation, regardless of sex.

### Sex-specific cytokines and chemokines in LC patients

**Figure 3:**
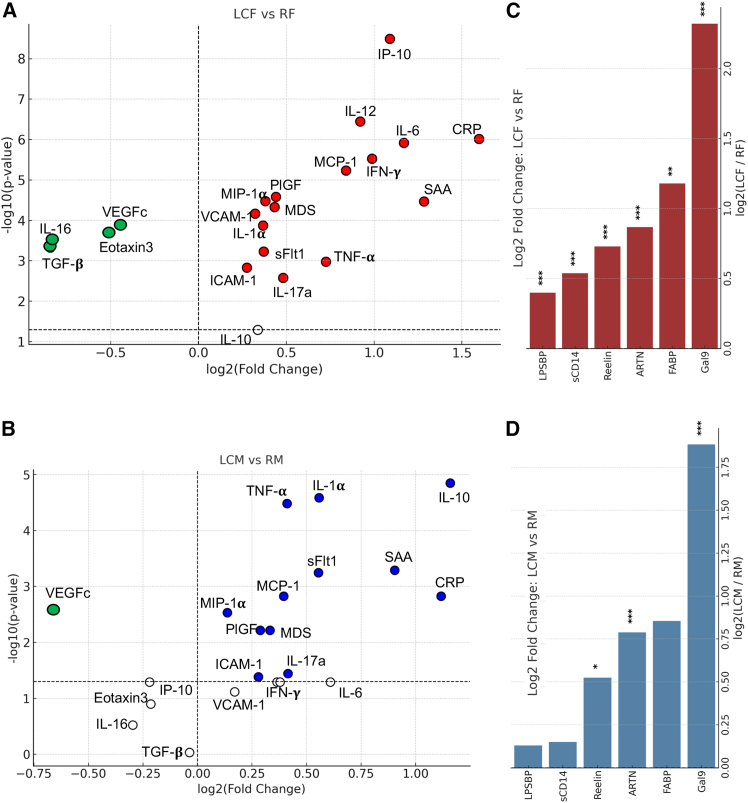
Sex-associated differential patterns of proinflammatory cytokines and chemokines in LC patients (A) Volcano plot of significantly elevated (red symbols) or reduced (green symbols) plasma biomarkers in LCF vs. RF. (B) Volcano plot of significantly elevated or reduced plasma biomarkers in LCM vs. RM. (C and D) (C) Comparison of plasma Gal9, I-FABP, ARTN, Reelin, sCD14, and LPSBP in LCF vs. RF (D) and LCM vs. RM. Data are shown as –log_10_(p-value) from unpaired t tests. ∗*p* < 0.05, ∗∗*p* <0.01, ∗∗∗*p* <0.001. Biomarkers with empty circles are not significantly different.

We found that pro-inflammatory cytokines/chemokines, including interleukin (IL)-1α, IL-6, IL-12/IL-23p40, inducible protein 10 (IP-10), interferon gamma (IFN-γ), tumor necrosis factor alpha (TNF-α), IL-17a, placental growth factor (PlGF), monocyte chemoattractant protein 1 (MCP-1), macrophage inflammatory protein 1α (MIP-1α), macrophage-derived chemokine (MDC), sFlt-1, VCAM-1, ICAM-1, C-reactive protein (CRP), and serum amyloid A (SAA) were significantly elevated in LCF compared to the RF group (Figures 3A, S2, and S3A–S3F). In contrast, transforming growth factor β1 (TGF-β1), IL-16, eotaxin-3, and vascular endothelial growth factor C (VEGF-C) levels were significantly reduced in LCF vs. RF (Figures 3A and S3A–S3D). In LCM, a limited number of cytokines/chemokines were elevated compared to the RM, including IL-1α, IL-10, TNF-α, IL-17a, PlGF, MCP-1, MIP-1α, MDS, ICAM-1, sFlt-1, CRP, and SAA (Figures 3B, S2, and S3A–S3F). However, only VEGF-C was significantly reduced in LCM vs. RM (Figures 3B and S3A–S3D). The elevation of IL-10 suggests a stronger anti-inflammatory signaling in the LCM cohort, but reduced TGF-β1 points to a weakened immunoregulatory capacity, potentially associated with reduced Tregs and persistent inflammation in LCFs.

### Sex-specific elevation of biomarkers associated with compromised gut integrity in LC patients

We have previously reported that compromised gut integrity may be associated with microbial translocation and persistent immune activation in LC patients.^16^ Therefore, we decided to determine whether sex plays a role in this phenomenon. Our analysis revealed a significant elevation in the levels of plasma I-FABP, LPS-BP, and sCD14 in LCF vs. RF but not in LCM compared to RMs (Figures 3C, 3D, and S3G–S3I). Additionally, we found significant elevation of Gal-9, ARTN, and Reelin in the plasma of both LCF and LCM patients, and the LCF cohort exhibited significantly higher levels compared to their male counterparts (Figures 2C, 2D, and S3J–S3L). Notably, the plasma levels of I-FABP, LPS-BP, Gal-9, ARTN, and Reelin were significantly higher in LCF than all other groups (Figures S3G–S3L).

### Sex hormone dysregulation in both male and female LC patients

**Figure 4:**
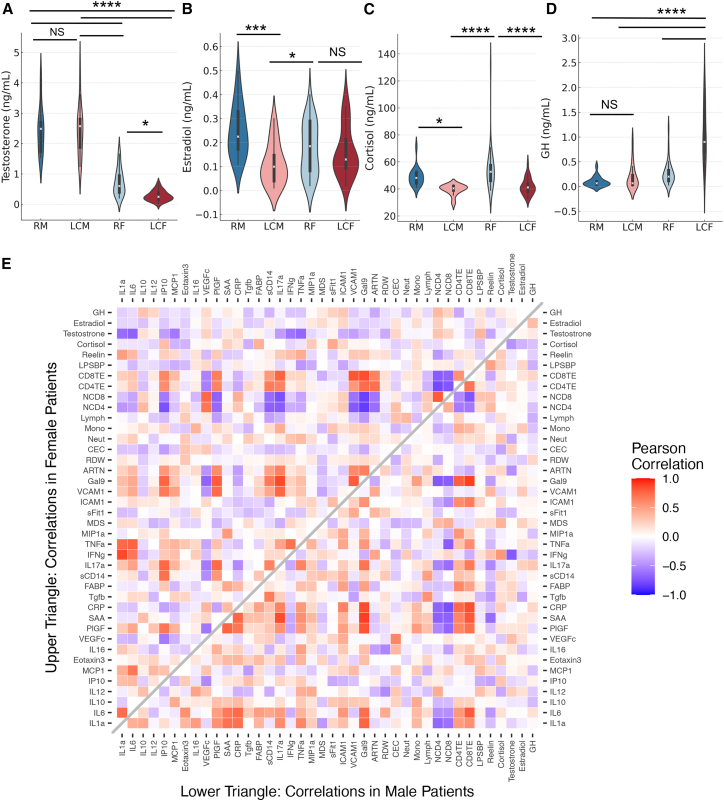
Differential levels of sex hormones in LCF vs. LCM (A–D) (A) Cumulative data of plasma testosterone; (B) estradiol; (C) cortisol; (D) growth hormone. (E) The heatmap of Pearson correlation of standardized parameters for LCF and LCM. *p* values were calculated using Kruskal-Wallis analysis with Dunn’s multiple comparisons test (A–D). Error bars represent mean ± SD. ∗*p* < 0.05, ∗∗∗*p* <0.001, ∗∗∗∗*p* <0.0001. ns, not significant.

We subjected plasma samples to analysis of testosterone, estradiol, progesterone, cortisol, growth hormone (GH), and T3 and T4 hormones. These analyses revealed a significant reduction in testosterone levels in LCF vs. RF, with no changes observed in testosterone levels in LCM vs. RM (Figure 4A). In contrast, estradiol levels were significantly reduced in LCM individuals when compared to RM (Figure 4B). Intriguingly, cortisol levels were substantially lower in both male and female LC patients (Figure 4C). Notably, plasma levels of GH were significantly elevated in LCF compared to other groups (Figure 4D). However, we did not observe any substantial differences in progesterone, T3, and T4 levels or the body mass index (BMI) between the groups (Figures S3M–S3P). Our findings suggest that LC patients may suffer from a relative deficiency in specific sex hormones, coupled with lower cortisol levels. These dysregulated hormonal levels may contribute, at least in part, to the differential immune signatures observed.

### Standardized correlational analysis reveal differences in LCF and LCM groups

**Figure 5:**
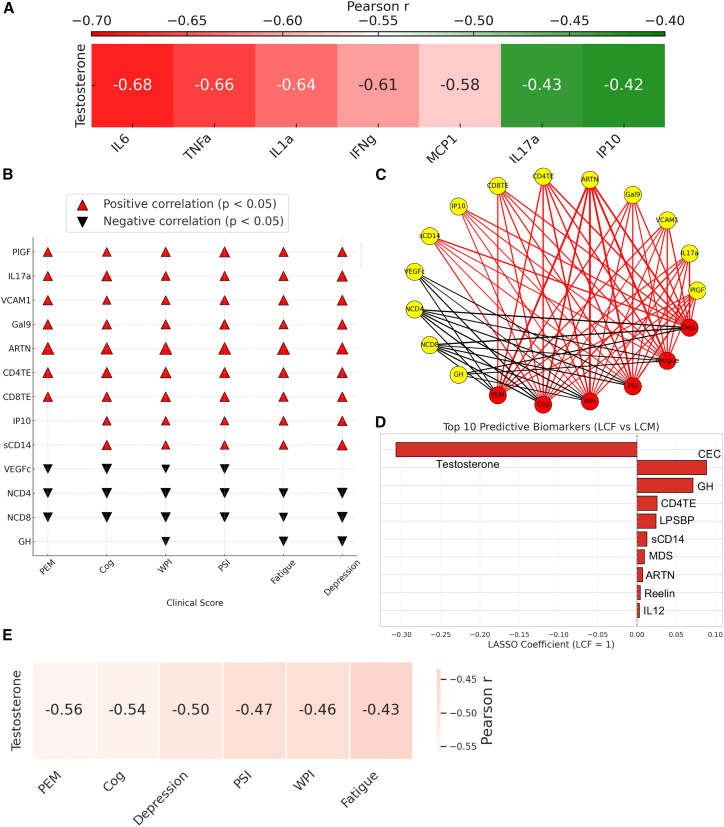
Standardized correlational analysis between biomarkers and clinical symptoms in LCF group (A) The inverse correlation between testosterone levels with various biomarkers in LCF using Pearson correlation analysis. (B) Bubble plot of significant correlations between plasma biomarkers and clinical symptom scores in LCF. Bubble size reflects the strength of the correlation (r), using Pearson correlation analysis. (C) Circular correlation network showing significant associations between plasma biomarkers (yellow) and clinical symptom scores (red) in LCF. Edge thickness reflects the strength of correlation; red and black edges indicate positive and negative correlations, respectively. (D) Top 10 predictive biomarkers distinguishing LCF from LCM based on LASSO regression. (E) The inverse correlation between testosterone levels and clinical scores in LCF. PEM, post-exertional malaise, PSI, pain severity index, WSP, widespread pain index, Cog, cognitive impairment.

We calculated Pearson correlations between 40 standardized parameters. The strength of correlations between parameters revealed physiologically expected and unexpected correlations. For example, we found that the frequency of CD4/CD8 TE was positively correlated with the plasma levels of ARTN, Gal-9, VCAM-1, sCD14, IL-17a, PlGF, and IP-10 in LCFs (Figure 4E). Similar analysis revealed that the frequency of CD4/CD8 TE was positively correlated with the plasma levels of CRP, SAA, PlGF, Gal-9, ICAM-1, IL-6, and IL-1α in LCMs (Figure 4E). Another important observation was an inverse correlation between plasma testosterone levels with various biomarkers in LCF patients (Figure 4E). Given the testosterone-mediated immunosuppression properties,^33^ we performed additional analysis to determine whether such inverse correlations were statistically significant. These analyses revealed moderate inverse correlation between the plasma levels of testosterone with IL-6, TNF-α, IL-1α, IFN-γ, MCP-1, IL17a, and IP10 in LCFs (Figures 5A and S4A). Additionally, we found a weak but significant inverse correlation between the plasma levels of testosterone with Reelin, VCAM-1, and RDW in LCFs (Figure S4A). While the inverse correlation between the plasma testosterone levels with cortisol and ARTN was not significant, we observed an inverse strong correlation between testosterone levels with IFN-γ in LCMs (Figure S4B). We also found a positive correlation between Gal-9 with IP10, PlGF, sCD14, IL17a, and VCAM-1 as well as a milder correlation of Gal-9 with IL-1α, IL-6, and MCP-1 in LCFs (Figure 4E). In contrast, we found that Gal-9 levels were positively correlated with IL-6, IL-1α, PlGF, SAA, CRP, IL-17a, TNF-α, and ICAM-1 in LCM group (Figure 4E). Although ARTN did not show any strong correlation with other biomarkers in LCM patients, it was positively correlated with IP-10, PlGF, VCAM-1, and Gal-9 (Figure 4E). When similar analysis was performed, we observed a notable positive correlation between SAA and CRP with LPS-BP, neutrophils count, I-FABP, and TNF-α only in the RF group but not in the RM group (Figure S4C).

### Biomarkers and clinical symptoms

To explore the potential mechanistic links between immune, endothelial, hormonal alterations, and the clinical manifestations of LC, Pearson correlation coefficients were computed to assess the linear relationships between 40 plasma biomarker levels (Figure 4E) and clinical symptom scores (e.g., fatigue, PEM, WPI, PSI, cognition, and depression). These analyses enabled the identification of 13 biomarkers that exhibited significant relationships with clinical symptoms in LCFs (Figures 5B and 5C). However, the same analysis revealed the association of most of these biomarkers mainly with cognitive impairment and depression scores in LCM (Figures S5A and S5B). Regularized regression models (LASSO and Ridge) were further used to identify the most predictive biomarkers distinguishing LCF from LCM. LASSO identified biomarkers, such as low testosterone but high CEC, GH, and CD4TE, as the most discriminative, assigning non-zero coefficients only to these features (Figure 5D). Ridge regression, while retaining all features, showed consistent directional effects for the same top markers (Figure S5C). Notably, we found an inverse correlation between testosterone levels with clinical scores in LCFs (Figure 5E). Comparison of model coefficients revealed robust sex-specific hormonal and clinical differences in LCFs.

### Distinct transcriptional profile in LCF and LCM

**Figure 6:**
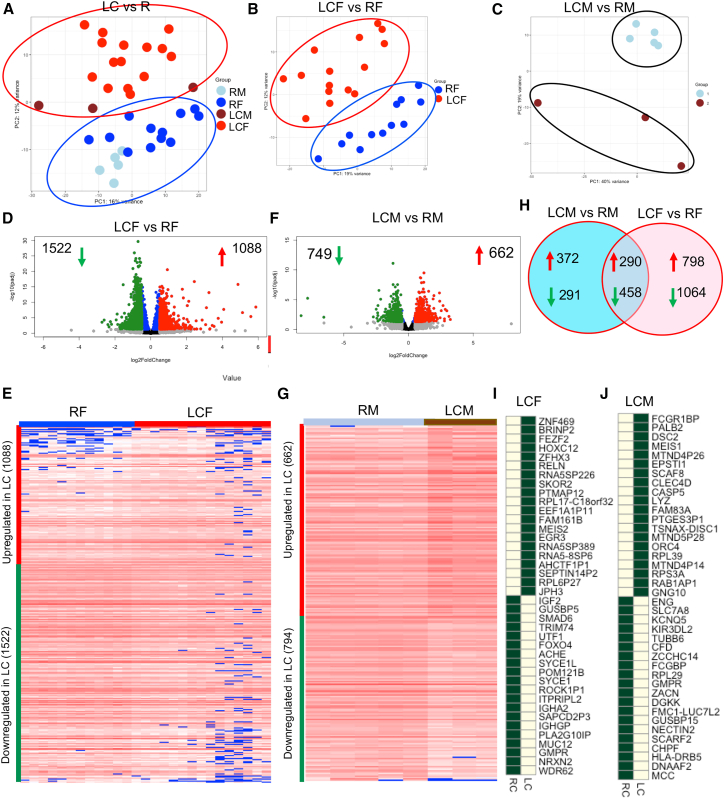
Male and female LC patients exhibit a distinct transcriptional profile (A) PCA on the Euclidean distances of the genes between whole blood of males and females within two study cohorts. (B) PCA on the Euclidean distances of the genes between whole blood of LC and RF. (C) PCA on the Euclidean distances of the genes between whole blood of LC and RM. (D) Volcano plot depicting the number and fold change (FC) of differentially expressed genes in whole blood of LCF vs. RF. The numbers of up- and down-regulated genes are noted in the right and left sides of each plot, respectively. (E) The heatmap demonstrates the normalized abundance of differentially expressed genes (*P*_adj_ < 0.05 and a log_2_ −1 < fold change [FC] > +1) in female groups. (F) Volcano plot depicts the number and fold change (FC) of differentially expressed genes in whole blood of LCM vs. RM. The numbers of up- and down-regulated genes are noted in the right and left sides of each plot, respectively. (G) The heatmap demonstrates the normalized abundance of differentially expressed genes (*P*_adj_ < 0.05 and a log_2_ −1 < fold change [FC] > +1) in LCM vs. RM. (H) The number of up- and downregulated genes in LCM vs. RM, LCF vs. RF, and common up- and down-regulated genes in both LCM and LCF vs. Rs. The heatmaps of the top 20 highly unregulated or downregulated genes in (I) LCF and (J) LCM.

We randomly selected 18 patients (15 LCF and 3 LCM) and 17 control individuals (12 RF and 5 RM) for bulk RNA-seq, with a comparable age range (median 47.65 ± 10:65 for LC and 48.15 ± 9.1 for R). Principal-component analysis (PCA) based on Euclidean distances revealed clear separation between LC and R (Figure 6A), LCF and RF (Figure 6B), and LCM and RM individuals (Figure 6C). Specifically, we identified 1,088 genes significantly upregulated and 1,522 genes significantly downregulated in LCF compared to RFs (Figure 6D), corroborated by differential gene expression patterns depicted in the heatmap (Figure 6E). When a similar analysis was performed, we found 662 genes significantly upregulated and 749 genes significantly downregulated in LCM compared to the RMs (Figure 6F), as indicated by differential gene expression patterns shown in the related heatmap (Figure 6G). Further analysis revealed that, of the significantly upregulated genes, 290 were common between LCF and LCM groups, and among the downregulated genes, 458 were common (Figure 6H). These findings highlight profound changes in gene expression patterns in LCF compared to LCMs.

### Gene expression associated with cognitive dysfunction and immune dysregulation in LCF patients

We analyzed the top 20 upregulated and downregulated genes in LCF versus RF and LCM versus RM groups. We excluded pseudogenes and genes with unknown functions. Importantly, most of the upregulated genes were associated with neuronal injury response and cognitive dysfunction. The most upregulated gene in LCF was the zinc-finger-containing gene (*ZNF469*; >6.7-fold), which is involved in neuronal differentiation^34^^,^^35^(Figure S6). Another significant upregulated gene, bone morphogenetic protein and retinoic acid (RA) inducible neural-specific protein 2 (*BRINP2*, >5.8-fold) (Figure S6), is associated with neurological development and disorders.^36^ The third most highly upregulated gene, forebrain embryonic zinc finger (*FEZF2*, >5.5-fold), is essential for deep-layer neuron development and function.^37^^,^^38^ *HOXC12*, another upregulated gene (>5.1-fold), is involved in hematopoietic stem cell (HSC) and hematopoietic stem and progenitor cell (HSPC) renewal and differentiation.^39^^,^^40^ Similarly, zinc-finger homeobox 3 (*ZFHX3*; >4.8-fold), which is involved in neuronal differentiation^34^^,^^35^ and extracellular matrix (ECM) remodeling,^41^ was upregulated in LCFs (Figure S6). *Reln* (>4-fold), an extracellular matrix protein (Reelin) influencing synaptic plasticity and cognitive function,^42^^,^^43^ which has been reported to be elevated at the gene and protein levels in LC patients,^17^ was upregulated in LCFs (Figures 6I and S6A). The *Reln* gene was also significantly upregulated in LCMs but fell below our cutoff value of 0.5 log2 fold (Figure S6B). Other upregulated genes include Ski family transcriptional corepressor 2 (*SKOR2*; >3.7-fold), linked to cerebellar Purkinje cells (PCs)^44^^,^^45^; *RPL17-C18orf32* (>3.4-fold), a ribosomal protein associated with Alzheimer disease^46^; and Meis homeobox 2 (*MEIS2*; >3.2-fold), associated with neuronal dysfunction and intellectual impairment,^47^^,^^48^^,^^49^ which were also highly upregulated in LCFs versus RFs (Figure 6I). Lastly, the early growth response 3 (*EGR3*; >3.1-fold), linked to neuropsychiatric disorders,^50^ was also elevated in LCFs (Figure 6I). In contrast, the most downregulated genes included WD40-repeat protein 62 (*WDR62*; > −2.7-fold), which is involved in neurodevelopment,^51^ Neurexin2 (*NRX2*; < −2.6-fold), which has been linked to neuropsychiatric disorders,^52^^,^^53^ and guanosine monophosphate reductase (*GMPR*; < −2.3-fold), affecting Rho-GTPase activity^54^ (Figure 6I). Another downregulated gene, the membrane-bound mucin 12 (*MUC12*; < −2.3-fold), is linked to ulcerative colitis (UC)^55^^,^^56^ (Figure 6I). The *SYCE1* gene encodes synaptonemal complex central element protein 1 (<−1.7-fold) and SYCE1L (<−1.6-fold), which are expressed in the synaptonemal complex of meiotic chromosomes and whose deficiency is linked to infertility in mice,^57^ both downregulated in LCFs (Figure 6I). Likewise, forehead box 04 (*FOXO4*; < −1.2-fold), another downregulated transcriptional factor involved in cell survival, proliferation, and apoptosis,^58^ was observed in LCFs (Figure 6I). None of the top 20 genes in LCF overlapped with the top 20 genes in LCM. Notably, our comparative RNA-seq analysis revealed that the top 20 upregulated genes in LCM were enriched for pathways related to persistent innate immune activation, interferon signaling, mitochondrial stress, and inflammation. In contrast, the top 20 upregulated genes in LCF were associated with neuroinflammation and cognitive dysfunction, suggesting both qualitative and quantitative differences in transcriptomic activation between the sexes.

### Gene expression associated with immune dysregulation, cellular stress, and tissue repair in LCM patients

The most upregulated gene in LCM was *FcγR1*-binding protein (>3.2-fold), which is involved in antibody-mediated immune responses, such as phagocytosis, antigen presentation, and immune complexe clearance^59^ (Figure 6J). The second most upregulated gene, partner and localizer BRCA2 (*PALB2*; >3.1-fold), is involved in DNA damage repair, and its mutation is associated with increased risk of male breast cancers.^60^ Desmocollin-2 (*DSC2*; >2.9-fold), which is involved in desmosome integrity and function, may lead to cardiac myopathy if it is dysregulated.^61^ Intriguingly, *MEIS2* (>2.8-fold), is commonly upregulated in both LCF and LCMs (Figure 6J). Epithelial-stromal interaction 1 (*EPSTI1*; >2.7-fold), which is involved in monocyte-endothelial cell adhesion, is associated with increased atherosclerosis plaques.^62^ The upregulation of the *SCAF8* gene (>2.6-fold) may influence the transition between elongation and termination in human cells.^63^ Another upregulated gene in LCM, *CLEC4D* (>2.5-fold), is a C-type lectin receptor mainly expressed by neutrophils and monocytes. It is involved in respiratory burst, phagocytosis, and production of inflammatory cytokines.^64^ Caspase-5 (*CASP5*; >2.4-fold) is one of the upregulated genes in LCM, which is involved in the inflammatory response upon microbial sensing, leading to nuclear factor κB (NF-κB) activation.^65^ Another interesting upregulated gene, lysozyme (*LYZ*; 2.4-fold), was notably upregulated (Figure 6J). In addition to its role in innate immunity, it elicits pain by activating TLR-4 during neuroinflammation.^66^ Another upregulated gene, Translin-associated factor X (*TSNAX-DISC1*), located upstream of Disrupted-in-Schizophrenia-1 (DISC1), has been associated with bipolar disorder (BP) and major depression disorder (MDD)^67^ (Figure 6J).

In contrast, we observed the downregulation of various genes associated with immune dysregulation, vascular and endothelial dysfunction, metabolic stress, and neuroimmune interactions. The Mutated in colorectal cancer (*MCC*) gene was the most downregulated (>−8.2-fold) and negatively regulates cell-cycle progression in epithelial and neuronal cells.^68^

The second most downregulated gene in LCM, Dynein axonemal assembly factor 2 (*DNAAF2*; > −7.7-fold), is involved in cilia formation and function, possibly linked to mucosal response.^69^ *HLA-DRB5* was highly downregulated (>−6.5-fold), suggesting dysregulated antigen presentation or autoimmune response^70^ in LCMs. Chondroitin polymerizing factor (*CHPF*; > −2.7-fold), a type II transmembrane protein, is involved in tissue remodeling and extracellular matrix synthesis, and its downregulation is associated with reduced glioma proliferation and migration.^71^ Scavenger receptor class F member 2 (*SCARF2*; > −2.4-fold), whose decline in plasma has been associated with chronic obstructive pulmonary disease,^72^ was also downregulated. The other downregulated gene, Nectin cell adhesion molecule 2 (*NECTIN2*; < −2.4-fold), is involved in cell adhesion, movement, differentiation, and survival^73^ in LCMs (Figure 6J). Other downregulated genes include Guanosine monophosphate reductase (*GMPR*; > −2.3-fold), influencing Rho-GTPase activity^54^; cellular ribosomal protein L29 (*RPL29*; > −2.1-fold), involved in protein synthesis^74^; the zinc-activated ion channel (*ZACN*; < −2.3-fold), involved in cellular signaling in human brain, thyroid, prostate, and stomach^75^; the potassium voltage-gated channel subfamily Q member 5 (*KCNQ5*; < −1.9-fold), mainly expressed in the central and peripheral nervous systems^76^; and *KIR3DL2* (<−1.9-fold), an inhibitory ligand associated with reduced IFN-γ and NK cell cytotoxicity.^70^

### Differential immune cell dysregulation in LCF and LCM patients

**Figure 7:**
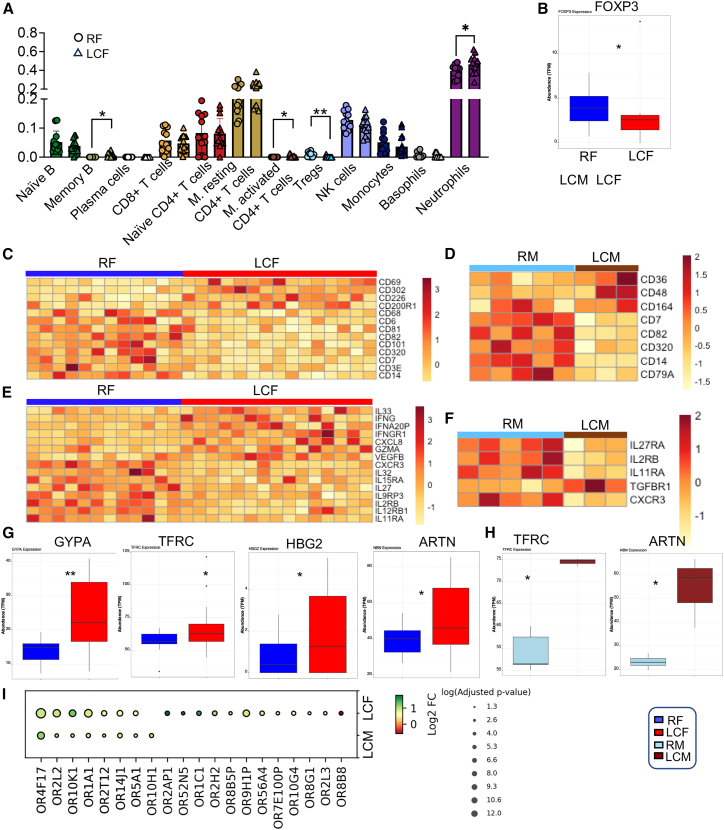
Sex-associated alteration in immune cell proportion in LC patients (A) Cumulative data using CIBERSORTx show the frequency of 12 immune cell types in LCF vs. RF. (B) Boxplot shows the abundance of FOXP3 in LCF vs. RF. (C and D) (C) Heatmaps representing upregulated and downregulated genes associated with immune cell markers in LCF vs. RF and (D) LCM vs. RM. (E and F) (E) Heatmaps representing upregulated and downregulated genes associated with various cytokines, chemokines, and their receptors in LCF vs. RF and (F) LCM vs. RM. (G) Boxplots depict the expression of different hematopoiesis-associated genes, such as glycophorin A (GYPA), transferrin receptor (TFRC), hemoglobulin G2 (HG2), and artemin (ARTN) gene in LCF vs. RF. (H) Boxplots depict the expression of TFRC and ARTN genes in LCM vs. RM. (I) Bubble plot of differentially expressed olfactory receptor (OR) genes in LCF vs. RF and LCM vs. RM. ∗*p* < 0.05, ∗∗*p* < 0.01.

To investigate the relative proportions of various immune cells in whole-blood RNA-seq data, we performed CIBERSORTx analysis to deconvolute the estimated 22 types of immune cells.^77^ A comparison of the relative frequencies of immune cell subsets between LCF and RFs revealed a significant increase in memory B cells, activated memory CD4^+^ T cells, and neutrophils, alongside with a significant reduction in Tregs (Figure 7A) in LCF. However, no significant differences were observed in the proportion of immune cells between the LCM versus RM (Figure S6C). Consistent with the CIBERSORTx analysis, we found a significant reduction in *FOXP3* gene expression in LCFs (Figure 7B). Additionally, several genes exhibited differential expression patterns in LCFs; *CD3e*, *CD14*, *CD7*, *CD320, CD101, CD82, CD81, CD6,* and *CD68* genes were significantly downregulated, whereas *CD69, CD302, CD226,* and *CD200R1* were significantly upregulated (Figure 7C). In contrast, in the LCM group, only *CD36, CD48,* and *CD164* genes were significantly upregulated, while *CD7, CD82, CD320, CD14,* and *CD79A* genes were significantly downregulated (Figure 7D). These findings highlight immune dysregulation in LC patients,^8^ underscoring a notable sex-associated difference in these patients.

### LC is associated with more pronounced modulation of cytokines, chemokines, and olfactory-associated genes in females

Given the observed immune dysregulation in LC patients,^8^^,^^78^ we analyzed the expression of differentially expressed pro-inflammatory cytokines, chemokines, and their corresponding receptors in RNA-seq data from our cohorts. These analyses revealed a significant increase in the expression of various genes, such as *IL-33, IFNG, IFNA20P, IFNGR1, CXCL-8, GZMA,* and *VEGF-B* in LCFs, compared to the controls (Figure 7E). In contrast, the expression of several cytokine/chemokine genes and their receptors, including *CXCR3, IL-32, IL-15RA, IL-27, IL-9RP3, IL-2RB, IL-12RB1,* and *IL-11RA*, were significantly downregulated in LCFs (Figure 7E). Interestingly, in LCMs, only *TGF-R1* was upregulated, while *IL-27RA, IL-2RA, IL-11RA,* and *CXCR3* were downregulated compared to the RM group (Figure 7F). Additionally, there was a significant increase in the expression of hematopoiesis-associated genes, such as glycophorin A (*GYPA*, CD235a), transferrin receptor (*TFRC*, CD71), hemoglobin G2 (*HBG2*), and *ARTN* in LCFs compared to the control group (Figure 7G). These observations align with our previous findings, which showed the expansion of CECs and elevated ARTN levels in the periphery of LC patients^8^^,^^16^ similar to what has been reported in acute COVID-19 disease.^28^^,^^29^^,^^79^ Although CD71 and ARTN were upregulated, we did not observe the upregulation of GYPA in LCMs (Figure 7H). This is consistent with our flow cytometry data (Figures 2N and 2O), which revealed the expansion of CECs exclusively in LCFs. The upregulation of CD71 may indicate immune activation, as it is expressed by activated immune cells.^80^ Another intriguing observation was the upregulation of eighteen olfactory receptor (OR) genes and the downregulation of one OR gene in LCFs (Figure 7I). In contrast, only six of these ORs were upregulated in LCMs (Figure 7I). While LCM exhibited the upregulation of OR10H1, LCF upregulated 11 distinct ORs (Figure 7I). ORs are seven-transmembrane G-protein-coupled receptors (GPCRs) in the olfactory epithelium (OE), where they detect odorants.^81^ However, their role in immune cells and blood circulation remains to be explored.

### Upregulation of sex-specific hormonal receptors in LCF patients

Considering the modulation of sex hormones in LC patients, we investigated potential sex-associated changes in the expression of sex hormone receptors in our RNA-seq data. We found the upregulation of estrogen receptor alpha (*ESR1*), estrogen receptor beta (*ESR2*), estrogen-related receptor gamma (*ESRRG*), androgen receptor (*AR*), and glucocorticoid receptor (*NR3C1*) genes in LCF patients (Figure S6D). The upregulation of ER genes may indicate heightened sensitivity to estrogen signaling. This could influence the expression of inflammatory cytokines and chemokines, glucose metabolism, and cardiovascular and neural functions.^82^ Similarly, the upregulation of *AR* gene could suggest a role for androgen signaling, potentially intersecting with estrogen pathways in females. The upregulation of *NR3C1* gene could influence anti-inflammatory pathways and stress responses, and altered sensitivity may contribute to immune dysregulation. Lastly, the upregulation of *ESRRG* gene may reflect metabolic and mitochondrial dysregulation, which could modulate cellular stress responses.^83^ These observations could partially explain heightened immune dysregulation in LCFs.

### Upregulation of pathways associated with oxidative stress response, tissue repair, and neuroinflammation in LCF patients

Given the large number of modulated genes in our RNA-seq data, we performed pathway analysis to gain deeper insights into LC pathogenesis. Using IPA software, our analysis revealed the upregulation of 19 pathways in LCF patients. Eight of these pathways were only upregulated in LCF (Figure S7), and the remaining 11 pathways were upregulated in both LCF and LCM patients (Figure S8). Overall, these pathways were associated with the cell cycle, ribosomal RNA processing, amino acid metabolism, protein synthesis, TGF-β signaling, neuronal signaling, selenoamino acid metabolism, and metabolic adaptations. The upregulation of these pathways highlights a complex interplay of cellular processes, including enhanced protein turnover (protein ubiquitination), stress adaptation (EIF2 signaling, amino acid deficiency response), and immune modulation (JAK-STAT and TGF-β signaling). Key effects include increased energy production (respiratory electron transport), improved ribosome biogenesis and protein synthesis (rRNA processing, translation initiation/elongation), and more stringent regulation of mRNA and protein quality (NMD, ribosomal quality control). However, the upregulation of these pathways may lead to cellular stress, immune dysregulation, oxidative damage, or tissue remodeling, potentially contributing to the pathophysiology in LCFs.

### Downregulation of pathways associated with immune dysregulation, impaired tissue repair, and reduced immune response against infection in LCF patients

We observed the downregulation of 39 pathways in LCFs associated with various biological processes (Figure S9). Here, we highlight some key insights into their potential role in the pathophysiology of LCFs. For example, the downregulation of neutrophil degranulation, phagosome formation, integrin signaling, TNF signaling, and IFN-α/β signaling may impair immune responses against infections (e.g., SARS-CoV-2) or hinder tissue repair.^84^ Similarly, impaired macrophage alternative activation and CD27 signaling may affect the resolution of inflammation and T cell immunity.^85^ The downregulation of pathways such as myelination signaling and the RHO GTPase cycle could point to dysregulation of nerve cell communication and cytoskeletal remodeling.^86^ These changes could impair neuronal connectivity and never repair mechanisms. Additionally, the degradation of the extracellular matrix, downregulation of GPCRs, and osteoclasts signaling could reduce cell proliferation, impair tissue repair, and affect bone health. In particular, GPCRs can cooperatively influence neuropsychiatric disorders, either directly or via the immune system.^87^ Other downregulated pathways, including glycosaminoglycan metabolism, sphingosine-1-phosphate signaling, and histone modification signaling, could lead to impaired tissue repair and cell differentiation. Finally, the downregulation of apelin cardiomyocyte signaling and insulin-like growth factor transport suggests potential cardiovascular dysfunction and metabolic dysregulation.^88^ Overall, these downregulated pathways suggest a complex interplay of immune dysfunction, impaired tissue repair, and dysregulated immune responses in LCFs.

### Upregulation of pathways associated with cellular stress response, immune activation, tissue repair, and increased metabolic demand in LCM patients

When pathway analysis was performed, we noted the upregulation of 47 pathways in LCM compared to the control group. Notably, among these pathways, 36 were exclusively upregulated in LCMs (Figure S10A) and 11 were common with LCF patients (Figure S8). Pathways such as nonsense-mediated decay (NMD), eukaryotic translation (initiation, elongation, termination), ribosomal quality control, and SRP-dependent cotranslational protein targeting to the membrane suggest increased protein synthesis activity in response to stress or increased demand, potentially linked to inflammation and cellular repair mechanisms.^89^ Similarly, the upregulation of oxidative phosphorylation and respiratory electron transport signaling pathways suggests heightened mitochondrial activity and energy production, often observed in conditions such as immune activation and tissue repair.^90^ As anticipated, we observed the upregulation of various pathways associated with ongoing immune activation, including IL-1 family signaling, noncanonical NF-κB signaling, the TNFR2 non-canonical NF-κB pathway, C-type lectin receptors (CLRs), and major histocompatibility complex (MHC) class I-mediated antigen processing and presentation (Figure S10A). We also noted the upregulation of various cell signaling pathways, such as S phase, mitotic metaphase and anaphase, cell-cycle checkpoints, regulation of mitotic cell cycle, DNA replication pre-initiation, and DNA synthesis, indicating increased cell proliferation, potentially associated with regenerative responses. Similarly, upregulation of hedgehog ligand biogenesis, hedgehog “on” state, and hedgehog “off” state signaling pathways suggests active tissue regeneration^91^ and repair process in LCMs. Additionally, upregulation of response of EIF2AK4 (GCN2) to amino acid deficiency,^92^ cell response to hypoxia, and KEAP1-NFE2L2 pathways^93^ suggest adaptation to nutrient deprivation and oxidative stress, commonly observed in conditions such as chronic inflammation. Finally, the upregulation of pathways, such as protein ubiquitination pathway, deubiquitination, and neddylation indicate heightened cellular protein regulation,^94^ possibly related to inflammation and DNA damage repair (Figure S10A). Overall, these upregulated pathways in LCM patients suggest an active physiological response to cellular stress, immune activating, tissue repair, and high metabolic demand.

### Downregulation of pathways associated with impaired immune responses, diminished cellular repair mechanisms, and defective intracellular signaling

Our further analysis revealed the downregulation of only four pathways in LCM compared to the control group (Figure S10B). The downregulation of the effect of PIP2 hydrolysis, involved in phosphatidylinositol 4,5-bisphosphate (PIP2) hydrolysis, can impair calcium-dependent processes, including immune cell activation, muscle contraction, and neurotransmitter release.^95^

The other downregulated pathway, phagosome formation, can impair the ability of innate immune cells to clear infections and damaged tissue. This might contribute to chronic inflammation due to an inability to resolve inflammatory responses effectively. The CLEAR (coordinated lysosomal expression and regulation) pathway, which controls lysosomal biogenesis and autophagy via the transcription factor TFEB,^96^ is also downregulated in LCM patients. Therefore, its downregulation could result in impaired lysosomal function and autophagy, leading to the accumulation of cellular debris, dysfunctional organelles, and reduced cellular stress adaptation. This may exacerbate inflammatory responses and contribute to neurodegenerative diseases or metabolic dysfunction. Finally, the downregulation of nucleotide salvage pathway, which recycles nucleotides from degraded DNA and RNA for reuse in nucleic acid synthesis, can lead to a shortage of nucleotides, affecting DNA repair and replication. This may cause genomic instability, increased susceptibility to DNA damage, and impaired cell proliferation, particularly in rapidly dividing cells like immune cells. Overall, the downregulation of these pathways suggests a combination of impaired immune responses, diminished cellular repair mechanisms, and defective intracellular signaling in LCMs.

### Common up- and downregulated pathways in LCF and LCM patients

Intriguingly, we observed that only 11 signaling pathways were upregulated in both female and male LC (Figure S8). The upregulation of these pathways in both male and female LC patients suggests a shared systemic response characterized by cellular stress adaptation, enhanced metabolic activity, immune activation, and regulation of proliferation and growth. For example, the PTEN pathway and translational control highlight attempts to maintain a balance between growth and stress adaptation.^97^ These findings suggest that the patients are experiencing systemic stress or immune challenges, necessitating robust cellular responses to maintain homeostasis and support tissue function. However, phagosome formation was the only common downregulated pathway in both LCM and LCF groups. This may not only impair host defense but also trigger sterile inflammation.

## Discussion

Our study reveals notable sex-specific differences in immune responses in LC patients with ME/CFS, highlighting the complex interplay between immune dysregulation, hormonal changes, and tissue repair. We observed differential immune cell phenotypes, cytokine profiles, and gene expression signatures in LCF and LCMs, which provide insights into the role of sex in ME/CFS pathophysiology. Notably, our study revealed that female sex is a prominent risk factor for ME/CFS in LC patients, consistent with the higher risk of ME/CFS in females following other infections.^98^

We found that LCF patients exhibited a marked decrease in lymphocyte counts and a relative increase in neutrophils and monocytes, suggesting a shift toward myelopoiesis at the expense of lymphopoiesis. This finding is in line with the dysregulated hematopoiesis observed in acute COVID-19 and LC patients.^8^^,^^79^^,^^99^ The reduction in naive CD4^+^ and CD8^+^ T cells further supports the notion of persistent immune activation in these patients,^8^ potentially due to chronic persistence of SARS-CoV-2 antigens in tissues^6^ or hyperimmune activation and autoimmunity.^5^^,^^8^ Interestingly, while both male and female LC patients displayed an expansion of TE T cells, the degree of immune dysregulation in LCF was more pronounced, particularly with the reduction in Tregs. This may contribute to compromised immunoregulation in LCF, consistent with prior studies in other inflammatory conditions.^100^^,^^101^ Additionally, diminution of CD39^+^ may explain the loss of FOXP3 in the presence of inflammatory milieu in LCF patients.^102^ The expansion of CECs and RDW supports a dysregulated erythropoiesis in LCF patients^8^^,^^29^ and may, in part, explain the symptoms of tiredness and fatigue observed in these patients. Given the immunomodulatory properties of CECs,^26^^,^^27^ their expansion may influence T cell effector functions in LCF. Notably, the ability of CECs to secrete ARTN may,^8^^,^^24^ at least in part, account for the elevated levels of ARTN in LCF patients. While CECs are not expanded in LCM patients, the source of ARTN could be linked to endothelial and/or neural cells.^103^ The elevated plasma levels of Gal-9 and their direct correlation with SARS-CoV-2-associated neurocognitive disorders (SANDs) resemble the role of Gal-9 in HIV-associated neurocognitive disorders (HANDs),^19^^,^^104^ whereas ARTN levels are reduced in HIV infection.^16^

LCF patients had significantly elevated levels of various pro-inflammatory cytokines and chemokines, suggesting an exaggerated inflammatory response as reported in idiopathic ME/CFS patients.^105^ In contrast, LCM patients exhibited increased levels of a fewer and more balanced inflammatory profile, with IL-10 being elevated. It appears that LC females exhibit the upregulation of type 2 interferon signaling (e.g., IP-10, IFN-γ), while LC males display IL-1 signaling. These observations suggest that altered sex hormone and cortisol levels in LC patients may underlie differential immune signaling patterns, paralleling mechanisms seen in autoimmune diseases and chronic viral infections.^106^^,^^107^ The reduction in TGF-β1 levels in LCF further implicates an impaired immunoregulatory mechanism, exacerbating the inflammatory environment and possibly promoting tissue damage and immune dysfunction.

Elevated levels of I-FABP, LPS-BP, and sCD14 in LCF patients suggest compromised gut integrity and microbial translocation, which may exacerbate systemic inflammation, as reported in HIV infection.^108^^,^^109^ These findings are consistent with previous reports linking gut dysbiosis and microbial translocation to persistent immune activation in idiopathic ME/CFS.^110^ The pronounced elevation of these biomarkers exclusively in LCF further emphasizes the role of sex in inflammatory burden. This may suggest a greater tendency for SARS-CoV-2 to migrate to the gastrointestinal tract in a subset of females, possibly due a higher expression of ACE2/TMPRSS2 expression,^111^ which subsequently results in a leaky gut and microbial translocation. Additionally, we identified a subset of plasma biomarkers that had significant correlation with key clinical symptoms in LC patients. Notably, these correlations were more pronounced in LCFs compared to LCMs. The elevated levels of these specific biomarkers in LCFs may contribute to the greater severity of symptoms observed in this subgroup.

We found that LCFs exhibited significantly reduced testosterone levels, which was associated with heightened systemic inflammation. The observed inverse correlation between testosterone levels and clinical scores in LCF supports a potential neuroprotective, anti-inflammatory, or metabolic regulatory role for this hormone.^112^^,^^113^^,^^114^ In contrast, LCM patients had lower estradiol levels, which may contribute to an altered cytokine milieu and increase IL-1 signaling.^106^^,^^107^ These hormonal changes in LC-associated ME/CSF are significant considering the established hormonal dysregulation in idiopathic ME/CFS.^115^ Cortisol levels were also reduced in LC patients across sexes, suggesting a dysregulated stress response that may exacerbate fatigue, pain, and fibromyalgia.^107^^,^^116^ The cause of low cortisol remains unclear but may involve partial adrenal insufficiency, hypothalamic-pituitary-gonadal (HPG) axis disruption, altered glucocorticoid metabolism, or enhanced negative feedback.^117^

Through RNA-seq, we identified a distinct transcriptional profile in LCF and LCM, with genes linked to neuronal injury and cognitive dysfunction upregulated in LCFs. This includes genes such as *ZFN469*, *BRINP2*, and *Reln*, which have been implicated in neurodevelopment and cognitive functions.^34^^,^^35^^,^^36^^,^^42^^,^^43^ These findings support the notion that LC, particularly in females, may involve cognitive dysfunction, potentially contributing to the “brain fog” frequently reported by patients. In contrast, LCMs exhibited upregulation of genes such as *FCγR1-BP, PALB2, DSCC, EPSTI1, CASP5,* and *MCC* associated with immune responses, vascular function, and stress, with a notable downregulation of genes related to immune dysregulation and endothelial dysfunction, highlighting the differences in immune response between the sexes.^59^^,^^60^^,^^61^^,^^62^^,^^63^^,^^65^ Notably, our bulk RNA-seq revealed upregulation of ORs,^17^ which was more prominent in LCF patients. This finding is consistent with emerging studies suggesting ectopic expression and functional roles of ORs beyond the olfactory system.^118^ Therefore, its upregulation may reflect broader dysregulation in sensory and neuroinflammation^118^ in LC patients with ME/CSF.

Furthermore, we found the upregulation of pathways involved in cell-cycle regulation, amino acid metabolism, and protein synthesis, which suggest an adaptive response to cellular stress, likely as a result of ongoing inflammation or oxidative damage in LCFs.^119^^,^^120^

In contrast, the downregulation of neutrophil degranulation, phagosome formation, integrin signaling, macrophage alternative activation, and CD27 signaling pathways further supports the notion of defective immune resolution, which may exacerbate inflammation and hinder tissue repair.^84^^,^^85^^,^^121^ Additionally, downregulation of pathways involved in myelination, Rho GTPase signaling, and extracellular matrix degradation point to potential disruptions in nerve cell communication, cytoskeletal remodeling, and tissue integrity.^86^ These collective findings highlight a multifaceted immune and repair dysfunction in LCFs, with potential consequences for both immune defense and tissue regeneration.

In LCMs, we observed the upregulation of pathways, many of which are associated with cellular stress response, immune activation, and tissue repair. The upregulation of oxidative phosphorylation and respiratory electron transport suggests enhanced mitochondrial activity, a hallmark of conditions involving heightened metabolic demand.^122^^,^^123^ Additionally, pathways associated with immune activation, such as IL-1 family signaling and noncanonical NF-κB signaling, reflect a systemic inflammatory response, while cell-cycle-related pathways and hedgehog signaling suggest active tissue regeneration and repair in LCM patients.^91^

Overall, our study provides compelling evidence of sex-specific immune and hormonal dysregulation in LC patients with ME/CSF. The differential immune cell phenotypes, cytokine responses, and gene expression patterns observed in male and female LC patients underscore the complexity of this condition. The findings suggest that LC may represent a multifaceted disease with distinct pathophysiological mechanisms in males and females, which may have important therapeutic implications—particularly as sex hormones have been proposed as a potential stratification strategy for patients with ME/CFS.^124^ In the context of LC, dysregulated sex hormones may be related to multisystem disruption or other specific factors. Notably, commonly observed symptoms in LCFs, such as muscle pain, fatigue, cognitive impairment, PEM, and sleep difficulties, substantially overlap with those seen in perimenopause/menopause and should be taken into consideration. Although further validation in larger cohorts is needed, our findings suggest that correcting hormonal levels may represent a potential therapeutic strategy to alleviate LC/ME/CSF symptoms.

### Limitations of the study

This study has multiple limitations. First, the unequal sample sizes between male and female groups may introduce potential biases. To mitigate this issue, we employed DESeq2 to adjust for sample size imbalances. While these adjustments reduce bias, unequal group sizes remain a limitation. Future studies with more balanced cohorts would enable a more comprehensive understanding of transcriptional differences between LCF and LCM. Second, as this is a cross-sectional study, we were unable to perform longitudinal assessments, such as tracking immune or hormonal changes over time. All participants were recruited at approximately 12 months post-acute COVID-19 disease; however, we did not collect precise information on the onset of ME/CFS-like symptoms following acute infection. This limits our ability to relate immunological or transcriptomic profiles to the chronology of disease development.

While our pathway analysis identified key biological processes dysregulated in LCF and LCMs, future studies should incorporate single-cell RNA-seq to resolve cellular heterogeneity, along with functional assays to assess immune cell function. Bulk RNA-seq, as used here, reflects an average signal across multiple cell types and does not capture gene expression at the resolution of individual cells. Given the complexity of immune responses and tissue repair mechanisms, cell-type-specific investigations (e.g., in immune cells, neurons, and fibroblasts) could yield more precise insights into pathway regulation. There may also be unmeasured confounding variables influencing gene expression, including environmental factors, genetic background, and comorbidities. These factors could impact our findings and should be accounted for in future studies. Additional research, including functional genomics or animal models, will be needed to determine the causal roles of the altered pathways in disease development and progression.

## Resource availability

### Lead contact

Requests for further information or resources should be directed to the lead contact, Shokrollah Elahi (elahi@ualberta.ca).

### Materials availability

This study did not generate new unique reagents.

### Data and code availability

• All data relevant to the study are included in the article or uploaded as supplemental information.
• RNA-seq data are publicly available at the SRA portal of NCBI under the accession number GEO: GSE270045.
• This paper does not report original code.
• Any additional information required to reanalyze data reported in this work is available from the lead contact upon request.

## Acknowledgments

We thank our study volunteers for providing samples and supporting this work.

We also thank the Flow Cytometry Core at the University of Alberta. RNA-seq experiments were performed at the University of Alberta Faculty of Medicine & Dentistry Advanced Cell Exploration Core, RRID:SCR_019182, which receives financial support from the Faculty of Medicine & Dentistry, the Li Ka Shing Institute of Virology, Striving for Pandemic Preparedness – The Alberta Research Consortium, and Canada Foundation for Innovation (CFI) awards to contributing investigators. This work was primarily supported by a grant from the 10.13039/501100000024Canadian Institutes of Health Research (CIHR #174901) to S.E. and two grants from the 10.13039/501100017001Li Ka Shing Institute of Virology, University of Alberta, Canada (to M.O. and S.E.). S.S. is supported by a 10.13039/501100000024CIHR REDI Early Career Transition Award, and M.O. is supported by the Arthritis Society Career Development (STAR/IMHA award number 00049). We also acknowledge BioRender.com, which enabled the design of graphic abstract.

## Author contributions

S.S. performed most of the study, analyzed bulk RNA-seq data, and designed related figures. M.O. designed clinical study, patient’s recruitment, clinical assessment, questionnaires, and clinical data analysis/interpretation; provided RNA-seq data; and obtained funding and resources. H.S. contributed to data acquisition of RNA-seq. A.M. provided resources/samples. R.J.R. performed correlation and ridge regression analyses. J.W.C.T. recruited patients and provided advice. S.E. assisted in data analysis, interpretation, supervision, resources, and study design; designed figures; and wrote the manuscript. All authors read/edited the manuscript.

## Declaration of interests

The authors declare no competing interests.

## Declaration of generative AI and AI-assisted technologies in the writing process

During the preparation of this work, the author(s) used ChatGPT to improve readability of the method section. After using this, the authors reviewed and edited the content as needed and take full responsibility for the content of the publication.

## STAR★Methods

### Key resources table

**Table:** 

REAGENT or RESOURCE	SOURCE	IDENTIFIER
**Antibodies**		

Mouse anti-human CD3 (Clone SK7), kappa APC-eFluor™ 780	Thermo Fisher	Cat#47-0036-42; PRID: AB_10717514
Mosue anti-human CD4 (Clone RPA-T4), kappa PE-Cy5	BD Biosciences	Cat#555348
Mosue anti-human CD8 (Clone RPA-T8), kappa BUV395	BD Biosciences	Cat#740303; PRID: AB_2740042
Mosue anti-human CD39 (Clone TU66), kappa BV711	BD Biosciences	Cat#563680; PRID: AB_2738369
Mosue anti-human CD71 (Clone MA712), kappa FITC	BD Biosciences	Cat#555536
Mosue anti-human CD235a/b (Clone HIR2), kappa PE-Cy™7	BD Biosciences	Cat#563666; PRID: AB_2738361
Mosue anti-human CD25 (Clone M-A251), kappa Alexa Fluor® 700	BD Biosciences	Cat#555348; PRID: AB_2738361
Rat anti-human FOXP3 (Clone 150D/EA), kappa PE	Thermo Fisher	Cat# 12-5773-82; PRID: AB_465936
Mouse anti-human CD45RA (Clone HI100), kappa Super Bright™ 600	Thermo Fisher	Cat# 63-0458-42; PRID: AB_2688186
Mouse anti-human CD28 (Clone CD28.2), kappa FITC	Thermo Fisher	Cat# 11-0289-42; PRID: AB_10733610
Mouse anti-human CCR7 (Clone 3D12), kappa PE-Cy™7	BD Biosciences	Cat# 557648
LIVE/DEAD™ Viability/Cytotoxicity Kit	Thermo Fisher	LIVE/DEAD™ Viability/Cytotoxicity Kit

**Biological samples**		

Human peripheral blood mononuclear cells (PBMCs) and plasma	Long COVID paients and individulas recovered from acute COVID-19 without any symptoms	Recruited at the University of Alberta hospital

**Critical commercial assays**		

Human Galectin-9 DuoSet ELISA	R&D Systems	Cat#DY2045
Human TGF-β DuoSet ELISA	R&D Systems	Cat#DY240
Human Artemin DuoSet ELISA	R&D Systems	Cat#DY2589
Human CD14 Quantikine QuicKit ELISA	R&D Systems	Cat#QK383
Human FABP1/L-FABP DuoSet ELISA	R&D Systems	Cat#DY870-05
Human Reelin ELISA Kit	Novus	Cat#NBP2-82529
Human Growth Hormone (GH) DuoSet ELISA	R&D Systems	Cat#DY1067
V-PLEX Neuroinflammation Panel 1 Human Kit	Meso Scale Discovery	Cat#K15210D
6-plex Steroid/Thyroid Hormone Discovery Assay	Eve technologies, Calgary, Alberta	https://www.evetechnologies.com/product/steroid-thyroid-6-plex-discovery-assay-serum-plasma-samples/

**Deposited data**		

Raw RNA-seq data	NCBI	GEO: GSE270045

**Software and algorithms**		

FlowJo	BD Biosciences	Version 10.10
R version 4.2.0	R Development Core Team	https://www.r-project.org/
Discovery workbench 3.0 software	Meso Scale Discovery	https://www.mesoscale.com/en/products_and_services/software
Ingenuity Pathway Analysis (IPA)	QIAGEN	https://www.qiagen.com
CIBERSORTx	Alizadeh and Newman Lab	https://cibersortx.stanford.edu/
GraphPad Prism version 10.4.2	GraphPad Software, Inc. V10	https://www.graphpad.com/features
Biorender	https://www.biorender.com/	https://www.biorender.com/
Python	python.org	v13.12

### Experimental model and study participant details

#### Human study

A total of 140 human subjects were recruited for this study, including seventy-eight LC patients, 58 females (mean age 49.5 ± 10.74) and 20 males (mean age 49.50 ± 14.6). Sixty-two COVID-19 patients who had recovered (R) from the disease without any obvious symptoms and complications, including 42 females (mean age 45.60 ± 12.85) and 20 males (mean age 48.25 ± 9.8), were also recruited (Figure 1A). Approximately 60% of individuals were infected with the original SARS-CoV-2 (Wuhan) viral strain in 2020 and the remaining were primarily infected with the Delta or Omicron variants, which were confirmed by PCR at the University of Alberta Hospital, Edmonton. Both LC and R were recruited ∼12 months (371 ± 19 days LC vs. 370 ± 8.9 days R) after the onset of SARS-CoV-2 infection as reported elsewhere.^8^ All LC patients were recruited through the LC clinic at the University of Alberta Hospital, Edmonton. Similarly, age-sex-matched R participants were recruited in the same manner. In our original R cohorts,^8^ we had 16 males. To match the size of the LC male subset, an additional 4 R males were recruited, increasing our cohort size.

Our ME/CFS patients were selected from a large pool of patients exhibiting LC symptoms. This was conducted through a comprehensive evaluation process that involved clinical assessments, laboratory tests, and the administration of well-defined questionnaires, as we have previously described.^8^^,^^30^ It is important to note that the majority of the pool of LC patients did not present with ME/CFS, but rather exhibited other LC-related symptoms. We diligently selected those meeting the criteria of ME/CFS as outlined in the Canadian Consensus criteria (CCC) for ME/CFS and WHO.^125^

Participants were included if they were at least 12 months post-acute SARS-CoV-2 infection and met the diagnostic criteria for ME/CFS,^125^ using well-defined questioners as described before.^8^ Particularly, the DePaul Symptom Questionnaire (2)–Post-Exertional Malaise (DSQ-PEM) was used to evaluate (PEM) in our cohorts. DSQ-PEM) evaluates the frequency and severity of five core PEM-related symptoms. Each symptom was rated on a 5-point Likert scale for frequency (0 = none of the time to 4 = all of the time) and severity (0 = not present to 4 = very severe). A symptom was considered clinically significant if both frequency and severity scores were ≥2 (i.e., occurring at least half the time and of at least moderate intensity). Participants were classified as experiencing PEM if they met this threshold for at least one core item, in line with previously validated criteria.^8^^,^^125^ The composite PEM score, which integrates symptom frequency and severity, was significantly elevated in LCF relative to LCM (Figure 1C). To be able to make a comparison, a composite PEM score was calculated by summing the frequency and severity ratings across five PEM-related items from the DSQ-PEM. Higher scores indicate greater PEM burden. For example, if patients A had a PEM score of 6, compared to a score of 4 in patient B. A PEM score 6 might suggest moderate burden and a PEM score of 4 might suggest a milder PEM symptom. Similarly, fatigue frequency and severity were evaluated using fatigue-related items from the DSQ. This study was approved by the Human Research Ethics Board (HREB) at the University of Alberta with protocol # Pro00099502. A written informed consent form was obtained from all participants.

#### Inclusion and exclusion criteria

Our study participants (LC and R) were age-and sex-matched and considering that the majority of our patients (>80%) had a mild acute infection, confounding health conditions were only observed in a small number of LC cohorts (Figure 1B). Individuals with non-specific chronic symptoms deemed unrelated to LC, based on clinical assessment and medical history, were excluded. Additional exclusion criteria included a prior diagnosis of ME/CFS unrelated to COVID-19, comorbidities with overlapping symptoms, and chronic conditions such as HIV, HCV, HBV, cancer, or other debilitating diseases unrelated to SARS-CoV-2. However, we were unable to collect detailed clinical data on menstrual cycle phase, menopausal status, or contraceptive use. These factors can influence hormonal and immune parameters and may contribute to biological variability. We note, however, that none of the study participants were pregnant at the time of sampling. Future studies should incorporate comprehensive reproductive and hormonal histories to better interpret sex-based differences in LC pathogenesis.

#### Clinical tests

Complete blood count (CBC) was performed at the clinical laboratory of the University of Alberta hospital.

### Method details

#### Cell isolation and culture

Peripheral blood mononuclear cells (PBMCs) were isolated using Ficoll-paque gradient methods (GE Healthcare).^126^ Cell cultures were performed in RPMI 1640 supplemented with 10% FBS and 1% penicillin/streptomycin (Sigma-Aldrich).

#### Flow cytometry analysis

Fluorophore-labeled antibodies with specificity to human cell antigens were purchased mainly from BD Biosciences and Thermo Fisher Scientific. The following antibodies were used in this study: anti-CD3 (SK7), anti-CD4 (RPA-T4), anti-CD8 (RPA-T8), anti-CD45RA, anti-CCR7, anti-CD28, anti-CD95, anti-CD39 (TU66), anti-CD71 (MA712), anti-CD235a (HIR2), anti-CD25 (M-A251), and anti-FOXP3 (150D/EA). To exclude dead cells, live/dead staining (Thermo Fisher Scientific) was used. The entire flow cytometry analyses were performed on freshly isolated PBMCs. To prevent variations, we used consistent flow cytometry panels with the same gating strategy for all study subjects, and a minimum of 100,000 events was acquired for each cell subset according to our protocols.^127^^,^^128^ BD Biosciences CompBeads were used for compensation control. Following staining, cells were fixed and acquired on an LSRFortessa-SORP or Fortessa- X20 (BD Biosciences) and analyzed using the FlowJo software (version 10).

#### Cytokine and chemokine multiplex analysis and ELISA assays

Frozen plasma samples at −20/80°C were thawed and centrifuged for 15 min at 1500g followed by dilution for quantifying cytokine and chemokine profiles. The concentration of cytokines and chemokines was quantified using the V-PLEX Neuroinflammation panel 1 kit (K15210D-1) from Meso Scale Discovery (MSD).^129^^,^^130^ This kit quantifies IL-1α, IL-1*β*, IL-2, IL-4, IL-5, IL-6, IL-7, IL-8, IL-10, IL-12/IL23p40, IL-13, IL-15, IL-16, IL-17A, IFN-γ, TNF-*α*, TNF-β, Eotaxin-1, Eotaxin-3, MIP-1*α*, MIP-1*β*, Thymus- and activation-regulated chemokine (TARC), IP-10, MCP-1, MDC, MCP-4, Vascular endothelial growth factor (VEGF-A), VEGF-C, VEGF-D, endothelial-specific receptor, tyrosine kinase with immunoglobulin-like loops and epidermal growth factor homology domains (Tie-2), Flt-1, placental growth factor (PlGf), basic fibroblast growth factor (bFGF), serum amyloid A (SAA), C-reactive protein (CRP), ICAM-I and VCAM-1 according to the manufacturer’s instruction. Data were acquired on the V-plex Sector Imager 2400 plate reader. Analyte concentrations were extrapolated from a standard curve calculated using a four-parameter logistic fit MSD Workbench 3.0 software.

The plasma Gal-9 concentration was quantified using the ELISA kit (R&D, DY 2045), similarly, TGF-β (R&D, DY240), ARTN (R&D, DY 2589), intestinal fatty acid binding protein-1 (I-FABP) (R&D, Z-001), LPS-Binding protein (DY870-05), human growth hormone (DY1067), Reelin (Novus, NBP2-82529), and sCD14 (DC140) were subjected to ELISA as we reported elsewhere.^16^^,^^24^^,^^130^^,^^131^ Additionally, the 6-plex Steroid/Thyroid Hormone Discovery Assay (MilliporeSigma, Burlington, USA) was used to simultaneously measure hormone levels in plasma samples. Assays were performed by Eve Technologies (Calgary, Canada) using the Luminex 200 system (Luminex, Austin, TX, USA).

#### Library construction and sequencing

Total RNA was extracted from fresh whole blood and a total of 100 ng of isolated RNA was used for library construction. as we have previously reported.^17^ The generated data are publicly available from the SRA portal of NCBI under the Accession Number GSE270045.

#### Bioinformatic data analysis

Adaptor sequences were trimmed from raw fastq files with 123fastq prior to pseudoalignment with Kallisto^132^ with 100 permutations and bias correction for fragment alignment to the human cDNA database (GRCh38). Differential expression (DE) analysis was performed by analyzing count data using the DESeq2 R package (R version 4.2.0). Differentially expressed genes demonstrated −1 < log_2_ fold change (FC) > +1 and corrected *p*-value (*P*_adj_) < 0.05. Principal component analysis (PCA), heat maps, volcano plots, and boxplots were generated using R scripts. Bubble plots were generated with Python scripts incorporating the log fold change and adjusted *p*-value (*P*_adj_). Pathway analysis was perform using IPA software (QIAGEN, USA, https://www.qiagen.com/us), as reported before.^17^

#### Digital cytometry

CIBERSORTx (https://cibersortx.stanford.edu/) was used to deconvolute the fractions and abundances of immune cells from whole blood bulk RNA-Seq data as we reported before.^17^^,^^77^ The abundance of cells in each cell type was compared between the groups. The *p*-values <0.05 were considered statistically significant.

### Quantification and statistical analysis

The Wilks-Shapiro test assessed the distribution of data. The Mann-Whitney U-test was used for non-normally distributed data. Measures are expressed as mean ± SD and a *p-*values <0.05 was considered to be statistically significant. In violin plots, the middle line represents the median, the bottom line 1^st^ quartile, and the top line 3^rd^ quartile. No randomization was performed and no data points were excluded. Pearson correlations were calculated for pairs of standardized biomarkers for different groups using the glmnet package in R.^133^ To identify biomarkers that distinguish LCF from LCM, we applied two regularized regression models: LASSO (Least Absolute Shrinkage and Selection Operator) and Ridge regression using Python (v3.12). Both models were trained on standardized plasma biomarkers and immune cell frequency data using 5-fold cross-validation. Pearson correlation coefficients were used to assess correlation between biomarkers and clinical symptoms using Python (v3.12).

## Supplemental information

Document S1. Figures S1–S10 and Table S1

Document S2. Article plus supplemental information
