# Supplementary material for: Integrated immune, hormonal, and transcriptomic profiling reveals sex-specific dysregulation in long COVID patients with ME/CFS
Source: Cell Rep Med. 2025 Nov 7;6(11):102449. doi: 10.1016/j.xcrm.2025.102449 (PMC12711683; doi:10.1016/j.xcrm.2025.102449)
Supplement: Document S1. Figures S1–S10 and Table S1 [file mmc1.pdf]

Cell Reports Medicine, Volume *6*

Supplemental information

Integrated immune, hormonal, and transcriptomic profiling reveals sex-specific dysregulation in long COVID patients with ME/CFS

Shima Shahbaz, Mohammed Osman, Hussain Syed, Andrew Mason, Rhonda J. Rosychuk, Jan Willem Cohen Tervaert, and Shokrollah Elahi

S Figure 1

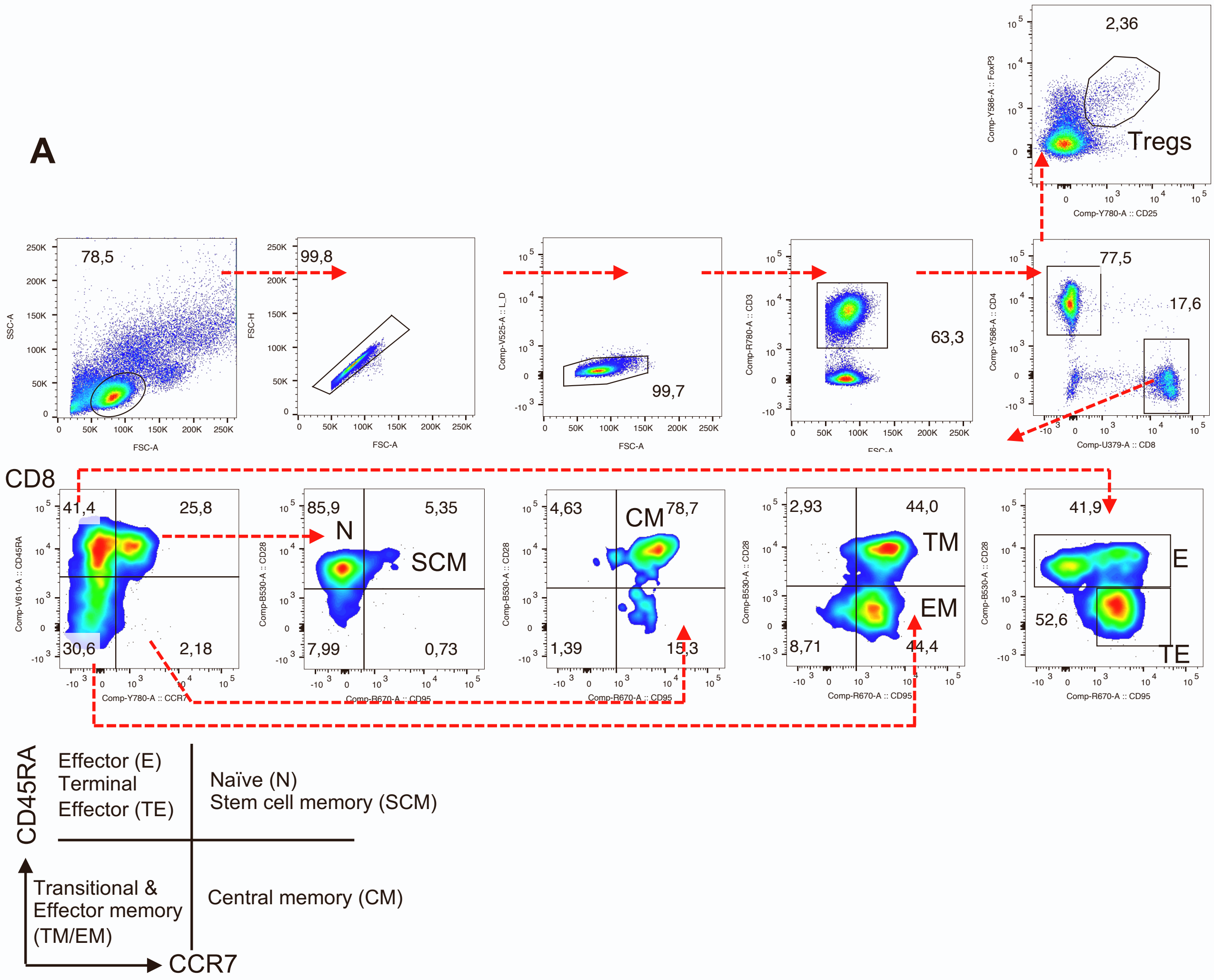

S Figure 1. Flow cytometry gating strategy, related to Figure 2. (A) The gating strategy of flow cytometry staining on peripheral blood mononuclear cells (PBMCs) for Tregs and different T cell subsets.

S Figure 2

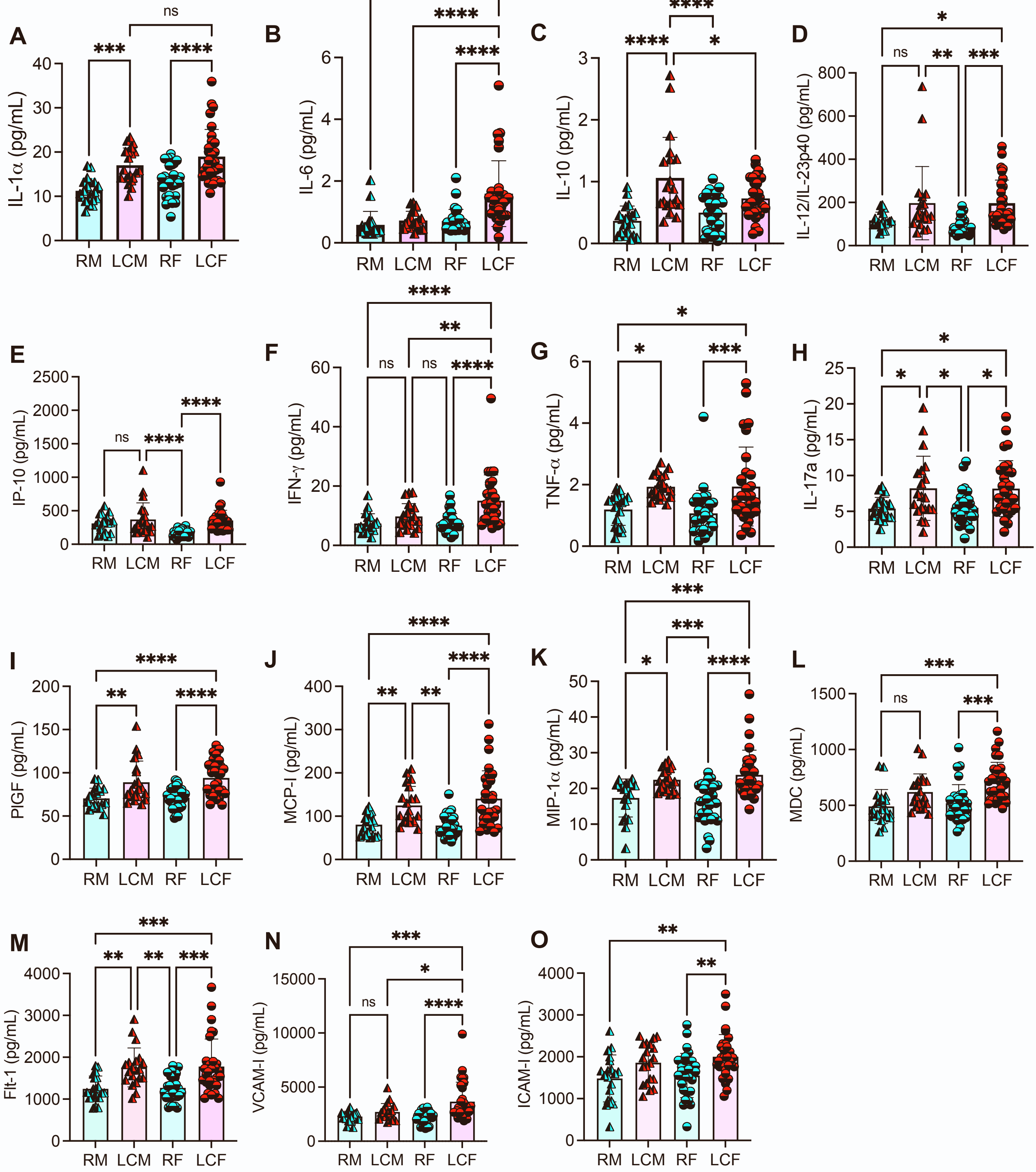

S Figure 2. Plasma cytokines and chemokines in LC and R groups, related to Figure 3. (A) Cumulative data of plasma IL-1α; (B) IL-6; (C) IL-10; (D) IL-12/23p40, (E) IP-10; (F) IFN-γ, (G) TNF-α; (H) IL-17a; (I) PlGF; (J) MCP-1; (K) MIP-1α; (L) MDC; (M) sFlit-1; (N) VCAM-1, and (O) ICAM-1 in different shown groups, measured by multiplex mesoscale (MSD) platform. *P* values were calculated using Kruskal–Wallis analysis with Dunn's multiple comparisons test (A-O). The blue symbol represents recovered (R) and red (LC). The circle and triangle symbols representing male and female, respectively. * P < 0.05, ** < 0.01, *** < 0.001, **** < 0.0001. Not significant (ns).

S Figure 3

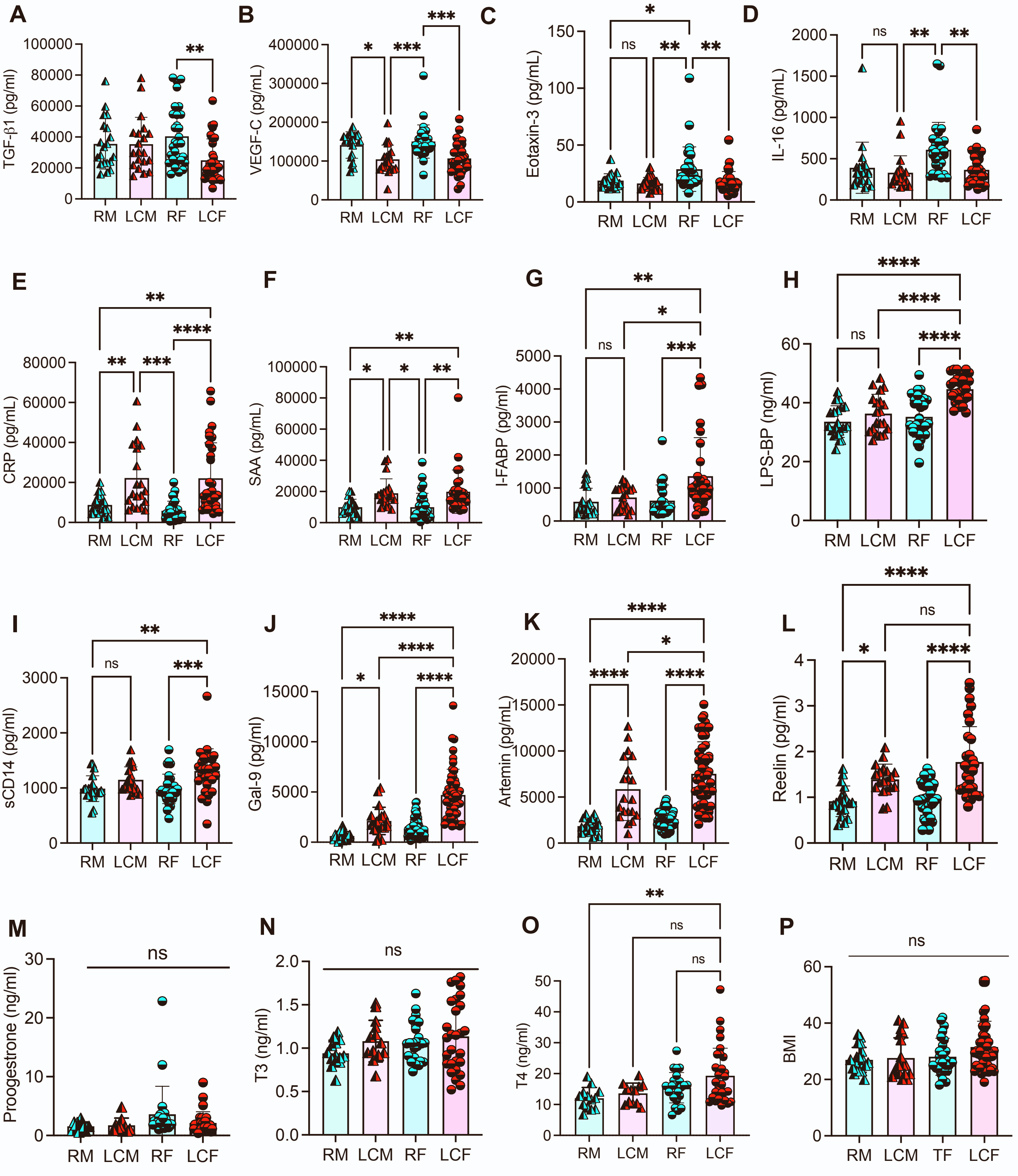

S Figure 3. Plasma cytokines, chemokines, and other biomarkers in LC and R groups, related to Figure 3 and Figure 4. (A) Cumulative data of plasma TGF-β1; (B) VEGF-C; (C) Eotaxin-3; (D) IL-16, (E) CRP, (F) SAA, (G) I-FABP; (H) LPS-BP; (I) sCD14; (J) Gal-9, (K) ARTN; (L) Reelin measured by multiplex mesoscale (MSD) platform or ELISAs. (M) Cumulative data of plasma levels of progesterone in different study groups. (N) Cumulative data of plasma concentrations of the thyroid triiodothyronine (T3), and (O) Thyroxine (T4) in different study cohorts measure by Luminex assay (Eve Technologies). (P) Cumulative data of body mass index in different study groups. *P* values were calculated using Kruskal–Wallis analysis with Dunn's multiple comparisons test (A-P). The blue symbol represents recovered (R) and red (LC). The circle and triangle symbols representing male and female, respectively. * P < 0.05, ** < 0.01, *** < 0.001, **** < 0.0001. Not significant (ns).

S Figure 4

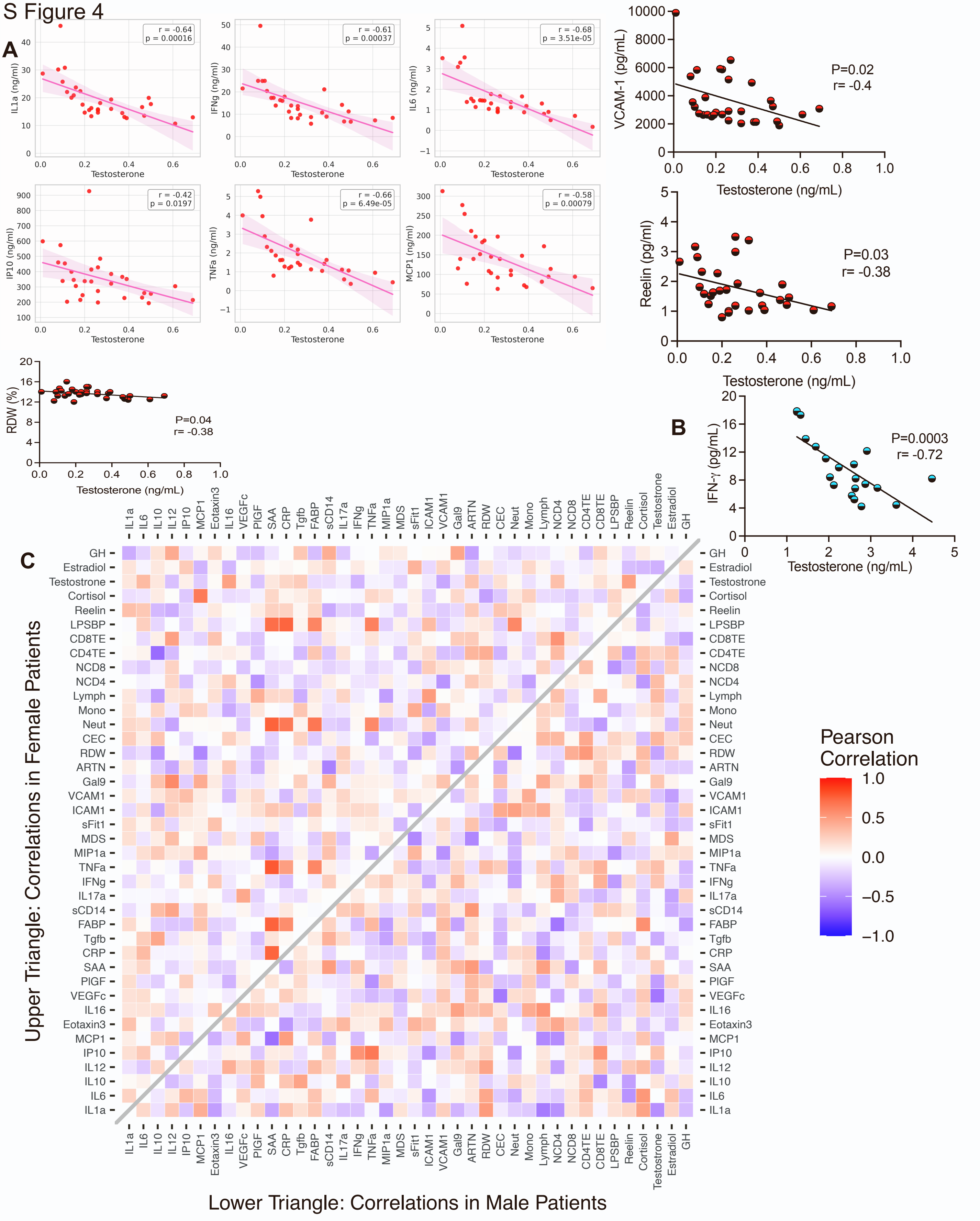

S Figure 4. Correlation of testosterone with plasma biomarkers and Pearson correlation of 40 biomarkers in RF vs RM groups, related to Figure 5. (A) Plots showing an inverse correlation between the plasma testosterone levels with various biomarkers in LCF patients. (B) The inverse correlation between the plasma testosterone levels with IFN-γ in LCM patients. (C) The heatmap of Pearson correlation of standardized parameters for RF and RM. Spearmen correlation analysis (A-C).

S Figure 5

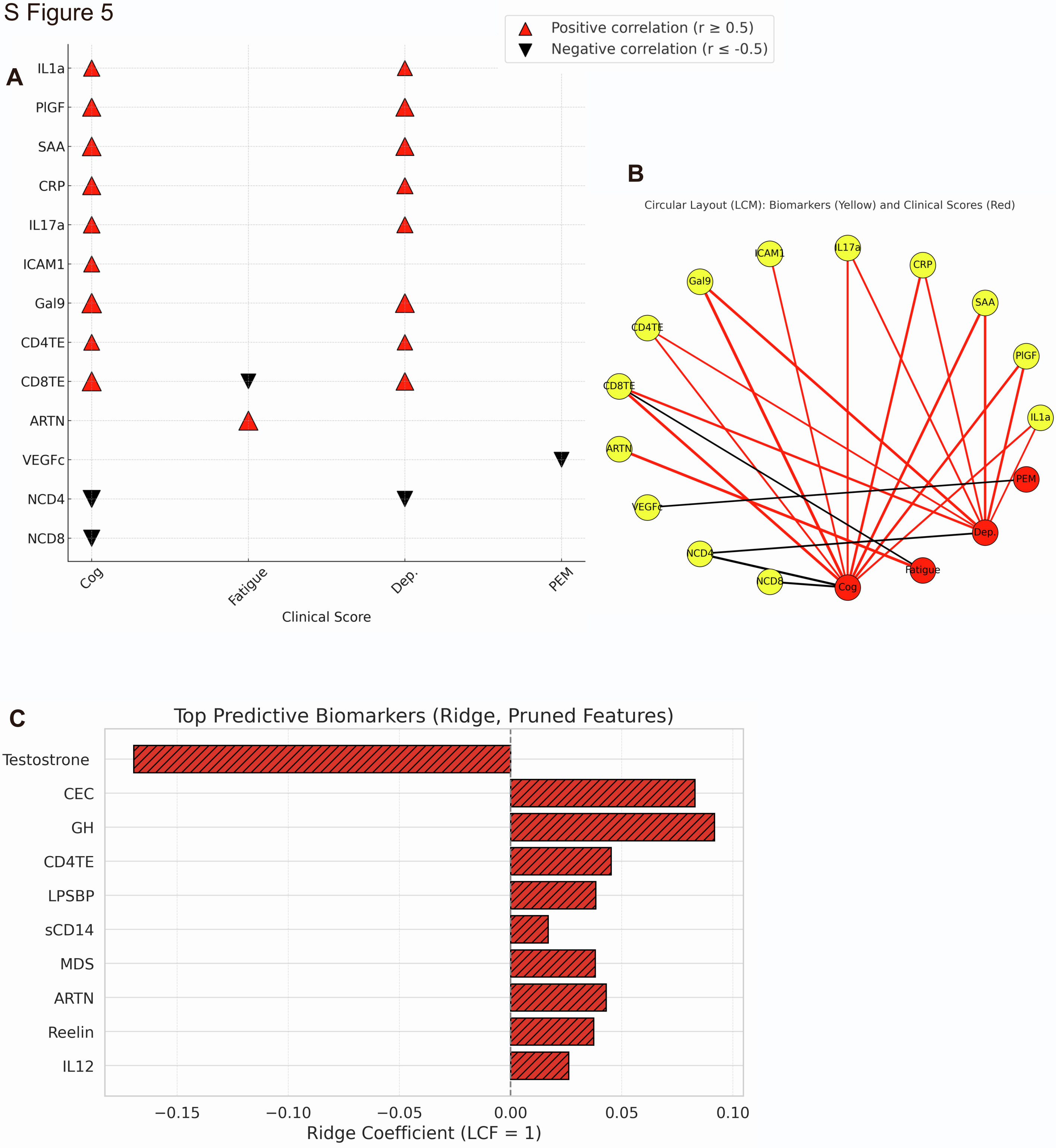

S Figure 5. Predictive biomarkers between LCF vs LCM and correlation of clinical symptoms with biomarkers in LCM patients, related to Figure 5. (A) Bubble plot showing significant correlations ($r \geq 0.5$, $p < 0.05$) between plasma biomarkers and clinical symptom scores in LCM. Upward red triangles indicate positive correlations; downward black triangles indicate negative correlations. Bubble size reflects the strength of the correlation (r), using Pearson correlation analysis. (B) Circular correlation network showing significant associations ($r \geq 0.5$, $p < 0.05$) between plasma biomarkers (yellow) and clinical symptom scores (red) in LCM. Edge thickness reflects the strength of correlation; red edges indicate positive correlations; black edges indicate negative correlations. (C) Top 10 predictive biomarkers distinguishing LCF from RF based on Ridge regression. Post-exertional malaise (PEM), Depression (Dep.), and cognitive impairment (Cog).

S Figure 6

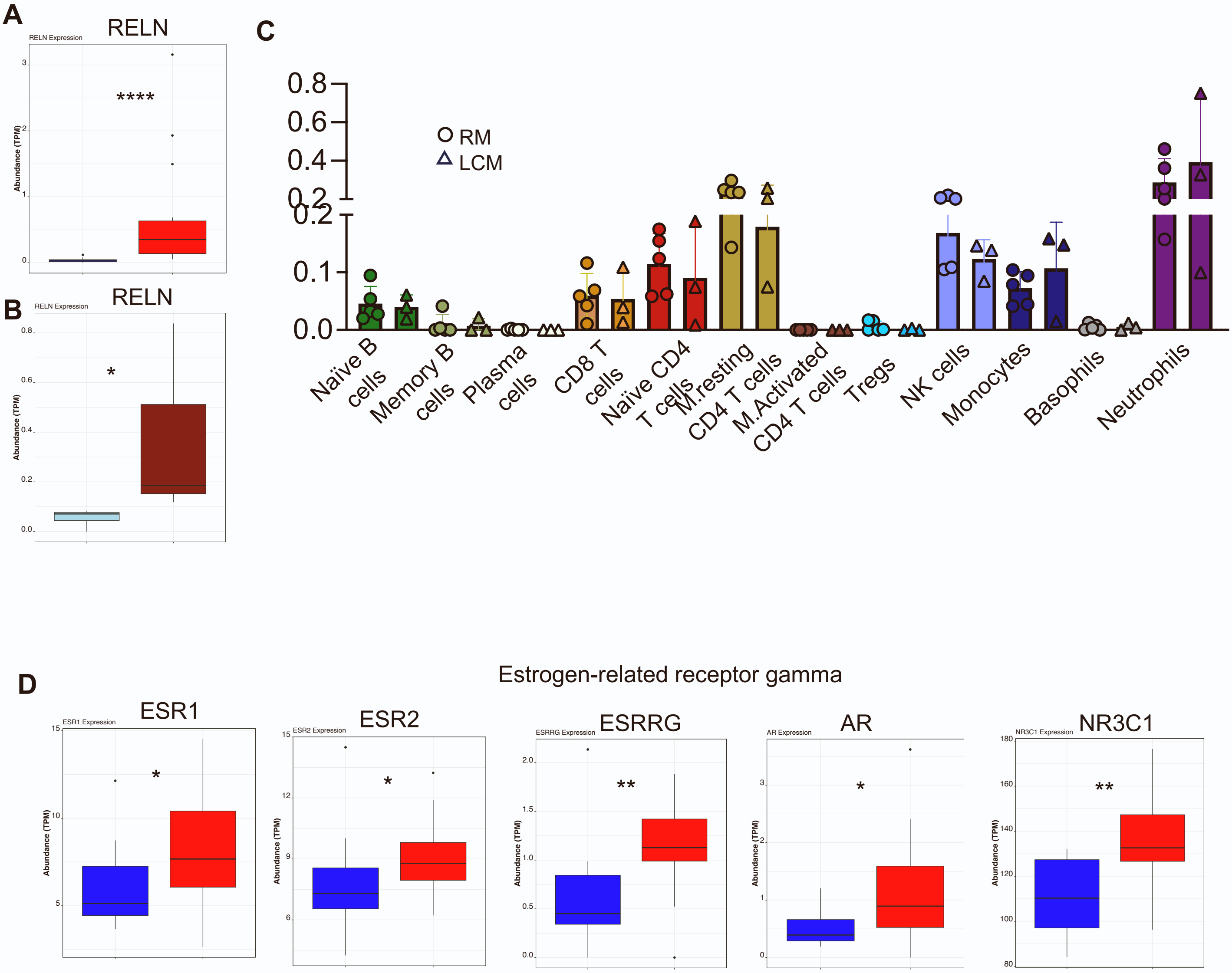

S Figure 6. CIBERSORTx analysis in LCM vs RM and differential expression levels of various genes, related to Figure 6. (A) Boxplots for *Reln* expression in LCF, and (B) LCM. **(C)** Cumulative data using CIBERSORTx shows the frequency of twelve types of immune cells in LCM patients vs male controls. (D) Boxplots depict the expression of significantly upregulated sex hormones estrogen receptor alpha (ESR1), estrogen receptor beta (ESR2), estrogen-related receptor gamma (ESRRG), and glucocorticoid receptor (NR3C1) genes in LCF vs RF.

S Figure 7

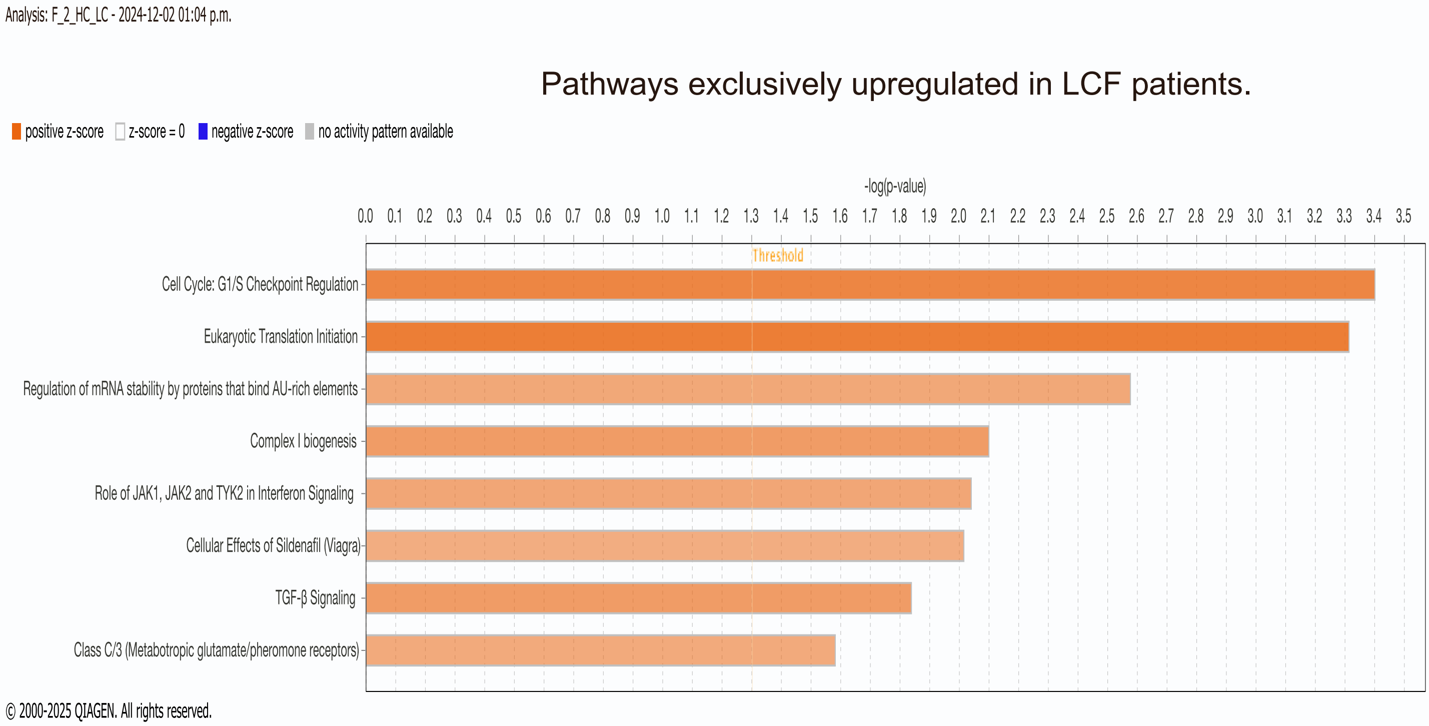

S Figure 7. Pathway analysis, related to Figure 6. Exclusively upregulated pathways in LCF patients.

S Figure 8

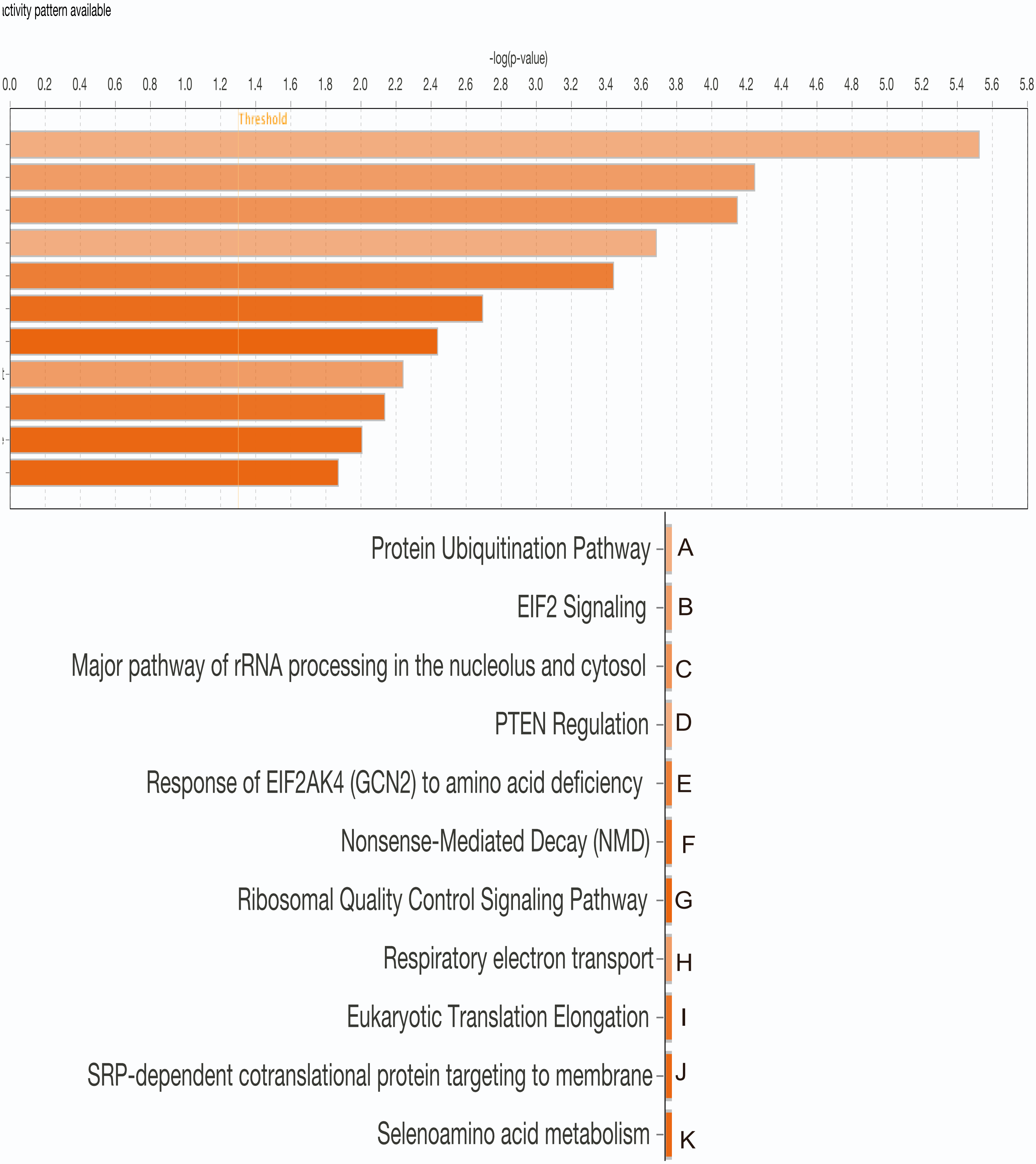

S Figure 8. **Pathways analysis, related to Figure 6.** The list of 11 commonly upregulated pathways in LCF and LCM patients.

S Figure 9

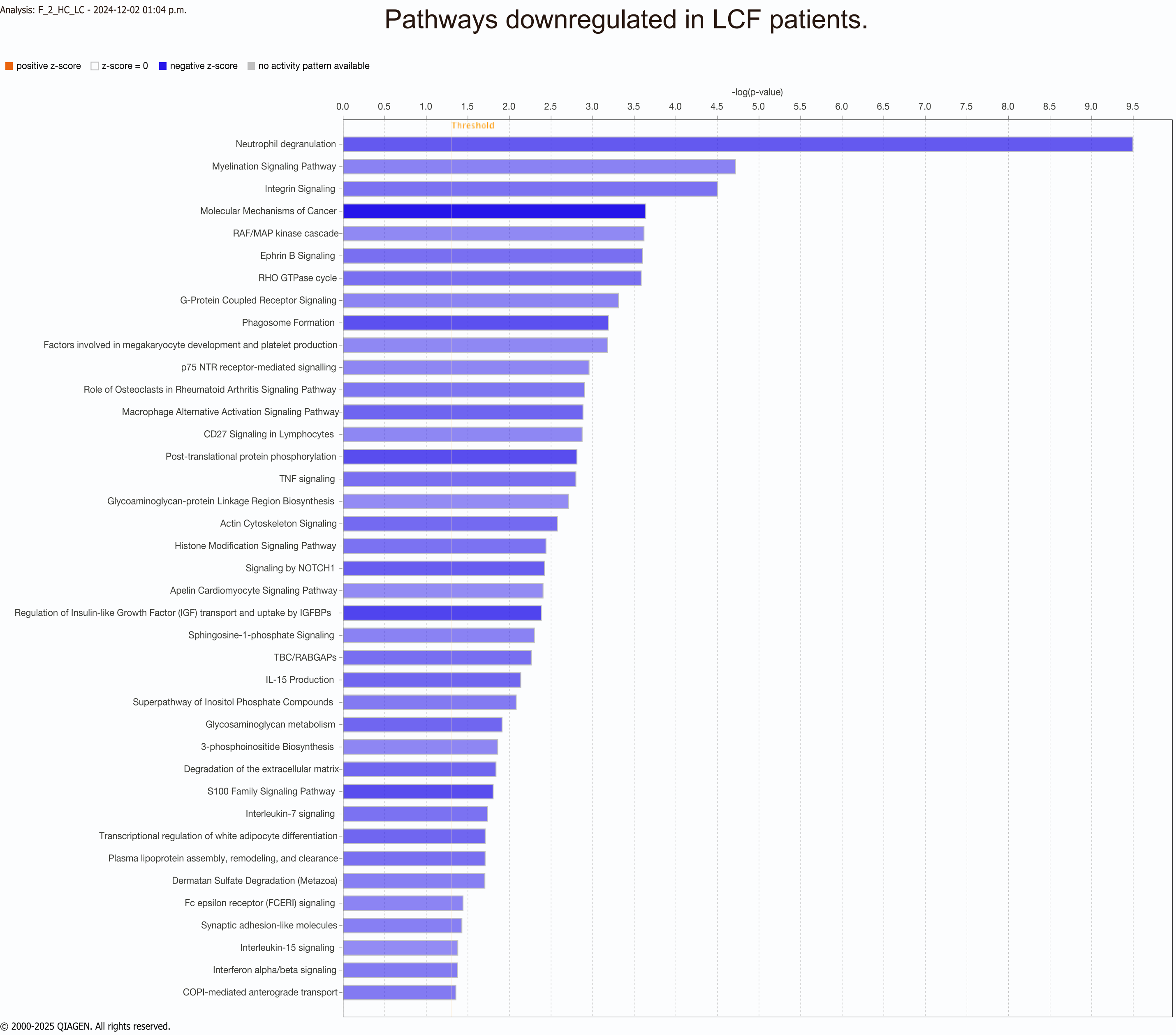

S Figure 9. Pathways analysis, related to Figure 6. The list of 39 downregulated pathways in LCF patients.

S Figure 10

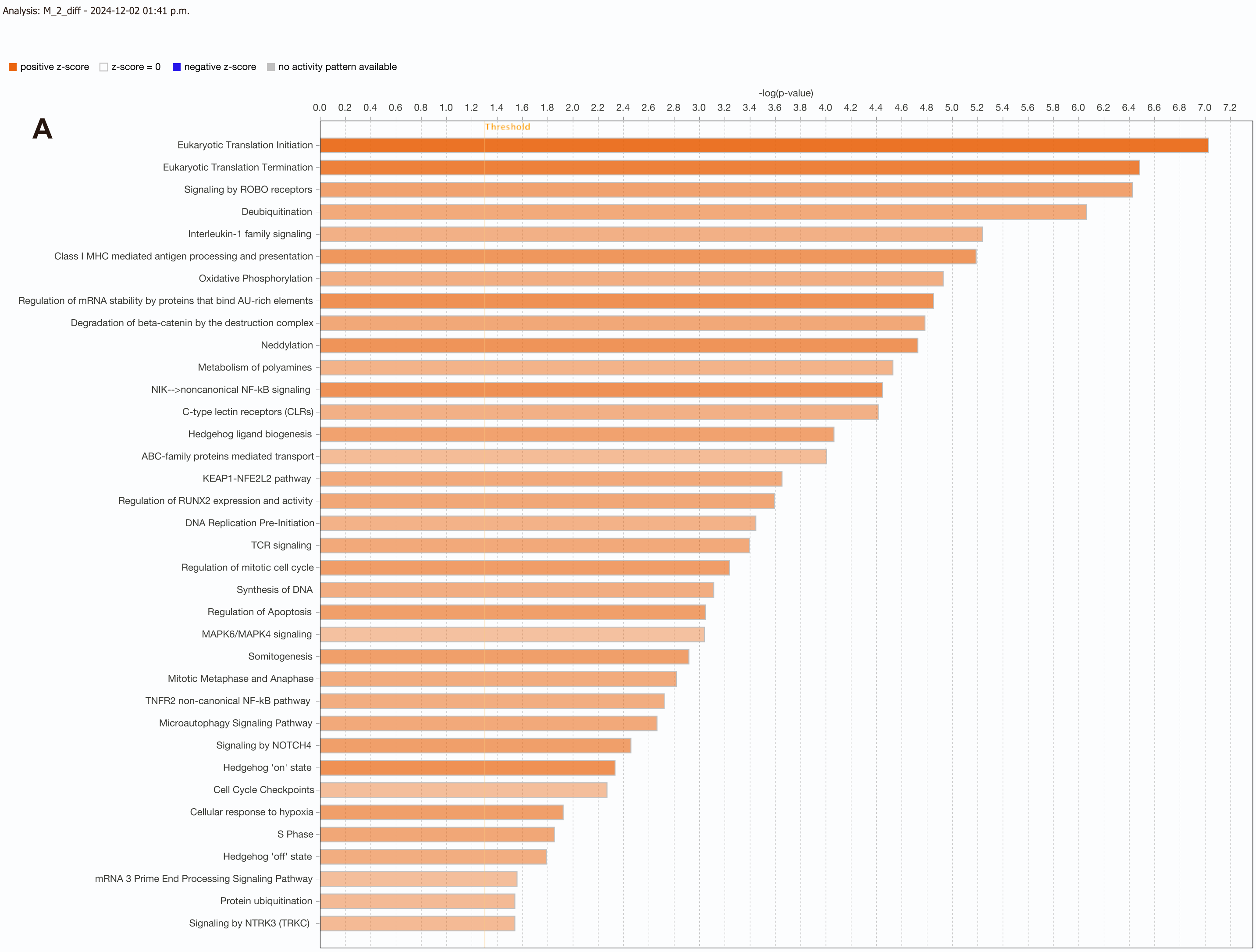

© 2000-2025 QIAGEN. All rights reserved.

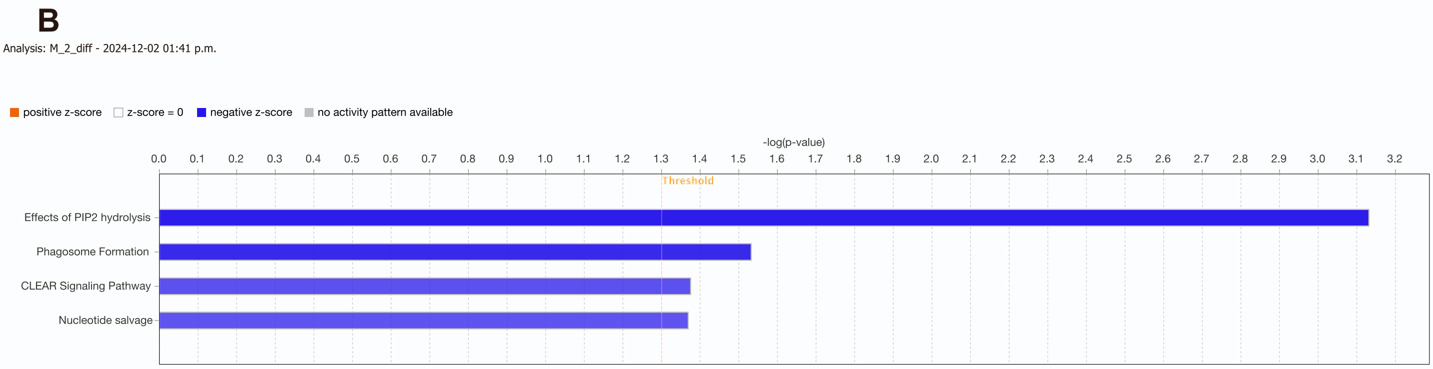

© 2000-2025 QIAGEN. All rights reserved.

S Figure 10. Pathway analysis, related to Figure 6. (A) The list of exclusively upregulated 36 pathways in LCM compared to the control group. (B) Downregulated pathways in LCM vs RM groups.

S Table 1. Demographic, clinical, CMV status, and sample collection timelines of our cohorts, related to Figure 1.

Patient ID	Age	Sex	Hospitalization	CMV status	PCR test date/initial sampling	Previous health issues	Time of post-COVID sampling
LC1	36	F	No	Post	11/05/2020		07/05/2021
LC2	63	F	Hospital ward	Neg	11/05/2020		15/06/2021
LC3	45	M	No	Neg	19/11/2020		22/11/2021
LC4	39	M	No	Neg	28/07/2020		25/07/2021
LC5	52	F	No	Post	07/05/2020		27/04/2021
LC6	74	F	No	Post	26/06/2020		10/06/2021
LC7	55	F	Hospital ward	Neg	07/07/2020		28/07/2021
LC8	51	F	Hospital ward	Post	25/07/2020		28/07/2021
LC9	48	F	ICU	Post	10/05/2020	Asthma	17/05/2021
LC10	47	F	No	Post	15/07/2020		28/07/2021
LC11	83	M	ICU	Post	10/10/2020		14/10/2021
LC12	57	M	No	Post	12/08/2020		5/08/2021
LC13	56	F	No	Neg	14/08/2020		9/08/2021
LC14	56	F	No	Neg	30/09/2020		23/09/2021
LC15	56	M	No	Post	26/06/2020	Asthma	31/06/2021
LC16	46	F	No	Post	12/08/2022		17/08/2021
LC17	61	M	No	Post	12/08/2020		17/08/2021
LC18	39	F	No	Neg	3/09/2020		19/09/2021
LC19	69	F	No	Neg	14/12/2020		30/11/2021
LC20	38	F	No	Neg	24/06/2020		29/05/2021
LC21	63	F	No	Post	18/11/2020		23/11/2021
LC22	38	F	No	Neg	30/06/2020		29/05/2021
LC23	38	F	ICU	Neg	27/10/2020		21/10/2021
LC24	82	M	Hospital ward	Neg	18/11/2020		29/11/2021
LC25	53	F	No	Neg	11/06/2020		17/05/2021
LC26	57	F	Hospital ward	Post	13/12/2020		07/12/2021
LC27	26	F	No	Post	29/12/2020		07/12/2021
LC28	47	F	No	Post	02/06/2020		10/06/2021
LC29	63	F	No	Post	30/11/2020	Inflammatory Arthritis	07/12/2021
LC30	43	F	No	Post	04/08/2020		08/08/2021
LC31	54	F	No	Neg	11/05/2020		17/05/2021
LC32	54	F	No	Neg	11/06/2020		08/05/2021
LC33	38	F	No	Post	19/11/2020		25/11/2021
LC34	59	F	No	Neg	28/07/2020		28/07/2021
LC35	33	M	No	Post	27/06/2020		10/06/2021
LC36	56	F	No	Post	26/06/2020		10/05/2021
LC37	44	M	No	Post	07/07/2020		17/07/2021
LC38	56	F	No	Neg	25/07/2020		29/07/2021
LC39	61	F	Hospital ward	Post	10/09/2020		17/09/2021
LC40	42	M	No	Post	15/07/2020		29/07/2021

S Table 1. Demographic, clinical, CMV status, and sample collection timelines of our cohorts.

LC41	38	F	ICU	Neg	10/06/2020		18/06/2021
LC42	26	F	No	Neg	12/07/2020		24/06/2021
LC43	39	F	No	Post	14/06/2020		29/05/2021
LC44	35	M	No	Post	11/06/2020		18/06/2021
LC45	48	F	No/No	Neg	07/09/2021	Lupus	25/10/2022
LC46	61	F	No/No	Post	11/09/2021		26/10/2022
LC47	48	F	No/Yes	Post	02/12/2021		28/11/2022
LC48	41	F	No/No	Post	13/10/2021		01/11/2022
LC49	59	F	No/No	Post	21/10/2021		01/11/2022
LC50	43	F	ICU/No	Neg	23/10/2021		02/11/2022
LC51	47	F	No/No	Post	02/11/2021		08/11/2022
LC52	44	F	No/Yes	Post	12/11/2021		15/11/2022
LC53	61	M	No/No	Neg	04/05/2021		17/11/2022
LC54	45	M	No/No	Post	06/11/2021		18/11/2022
LC55	41	F	Hospital ward/No	Post	09/09/2021		18/11/2022
LC56	39	F	No/No	Neg	11/10/2021		22/11/2022
LC57	56	F	H. ward/Yes	Post	25/09/2021		23/11/2022
LC58	55	F	No/No	Post	10/11/2021		28/11/2022
LC59	43	F	No/No	Post	21/10/2021	Overweight	30/11/2022
LC60	43	F	No/Yes	Post	14/10/2021	Migraine	01/12/2022
LC61	38	M	No/Yes	Post	05/11/2021		01/12/2022
LC62	50	F	No/No	Neg	01/11/2021		05/12/2022
LC63	30	M	No/No	Post	01/11/2021		07/12/2022
LC64	48	M	No/No	Post	11/11/2021		07/12/2022
LC65	38	M	No/Yes	Post	11/12/2021		09/12/2022
LC66	60	M	ICU/No	Post	16/04/2021	COPD	09/12/2022
LC67	50	M	No/No	Neg	15/04/2021	HBP	13/12/2022
LC68	43	M	No/No	Post	14/03/2021		13/12/2022
LC69	63	F	No/Yes	Post	18/08/2021		13/12/2022
LC70	56	F	No/No	Neg	04/12/2021		16/12/2022
LC71	43	F	No/No	Post	01/10/2021		19/12/2022
LC72	47	F	No/No	Neg	15/11/2020		19/12/2022
LC73	69	F	No/No	Post	08/10/2021	Diverticulitis	20/12/2022
LC74	53	F	No/No	Post	20/12/2021		20/12/2022
LC75	48	F	No/No	Neg	02/04/2021		20/12/2022
LC76	49	F	No/No	Neg	30/08/2021	Sarcoidosis	21/12/2022
LC77	65	F	No/No	Post	17/06/2020		22/12/2022
LC78	24	F	No/Yes	Neg	12/08/2021		22/12/2022

S Table 1. Demographic, clinical, CMV status, and sample collection timelines of our cohorts.

R1	33	M	No	Neg	01/06/2020		16/06/2021
R2	30	F	No	Pos	19/11/2020	Lupus	18/11/2021
R3	65	F	Hospital ward	Pos	01/09/2020	Reactive Arthropathy	10/09/2021
R4	34	F	No	Pos	12/08/2020		12/08/2021
R5	56	M	No	Pos	12/10/2020		18/10/2021
R6	28	F	No	Pos	02/07/2020		11/07/2021
R7	72	M	Hospital ward	Pos	10/08/2020		8/07/2021
R8	28	F	No	Neg	09/06/2020		15/06/2021
R9	50	M	No	Neg	30/11/2020		28/11/2021
R10	51	F	No	Neg	24/11/2020		28/11/2021
R11	35	F	No	Pos	09/06/2020		16/06/2021
R12	52	M	No	Pos	01/08/2020		11/08/2021
R13	61	F	No	Pos	17/12/2020		15/12/2021
R14	69	F	No	Neg	21/06/2020		11/06/2021
R15	54	F	Hospital ward	Neg	12/10/2020		18/10/2021
R16	50	F	No	Pos	02/06/2020		11/06/2021
R17	49	M	No	Pos	10/08/2020		12/08/2021
R18	51	F	No	Pos	19/07/2020		11/07/2021
R19	37	F	No	Pos	30/09/2020		25/09/2021
R20	39	F	No	Pos	24/10/2020		25/10/2021
R21	54	F	No	Pos	05/07/2020		08/07/2021
R22	68	F	Hospital ward	Neg	11/07/2020		18/07/2021
R23	55	F	No	Neg	05/06/2020		16/06/2021
R24	39	F	No	Neg	09/08/2020		06/08/2021
R25	57	M	No/Yes	Post	22/10/2021		25/10/2022
R26	46	F	No/Yes	Post	10/10/2021		25/10/2022
R27	31	F	No/Yes	Neg	01/09/2021		26/10/2022
R28	41	M	No/Yes	Post	12/08/2021		26/10/2022
R29	26	M	No/Yes	Post	12/10/2021		01/11/2022
R30	28	F	No/Yes	Post	05/08/2021		02/11/2022
R31	52	F	No/No	Post	10/10/2021	HBP	08/11/2022
R32	28	F	Hospital ward/No	Post	09/08/2021		15/11/2022
R33	55	F	No/No	Post	11/11/2021		15/11/2022
R34	51	M	No/Yes	Neg	24/11/2021		17/11/2022
R35	35	F	No/Yes	Post	28/09/2021		18/11/2022
R36	22	F	No/No	Post	04/10/2021		22/11/2022
R37	61	F	No/Yes	Neg	26/12/2021	HBP	22/11/2022
R38	38	M	No/Yes	Neg	11/07/2021		22/11/2022
R39	40	M	H. ward/Yes	Post	02/10/2021	Diabetes	28/11/2022

S Table 1. Demographic, clinical, CMV status, and sample collection timelines of our cohorts.

R40	52	M	No/Yes	Neg	27/09/2021		28/11/2022
R41	47	M	No/Yes	Neg	10/08/2021		30/11/2022
R42	53	F	No/No	Neg	17/07/2021		30/11/2022
R43	34	F	No/Yes	Post	13/09/2021	IBD	01/12/2022
R44	37	F	No/Yes	Post	23/10/2021		05/12/2022
R45	44	F	No/Yes	Post	15/09/2021		05/12/2022
R46	65	F	H. ward/Yes	Neg	08/11/2021		07/12/2022
R47	49	M	No/Yes	Post	25/11/2021		09/12/2022
R48	39	F	No/Yes	Post	19/11/2021		13/12/2022
R49	46	F	No/No	Post	30/11/2021		13/12/2022
R50	41	F	No/Yes	Post	22/11/2021		16/12/2022
R51	54	M	H. ward/Yes	Post	14/12/2021		16/12/2022
R52	36	F	No/Yes	Post	17/09/2021		19/12/2022
R53	64	F	No/Yes	Post	14/10/2021		19/12/2022
R54	37	F	No/No	Post	10/10/2021		21/12/2022
R55	44	F	No/ Yes	Post	25/11/2021		21/12/2022
R56	57	F	No/Yes	Post	06/09/2021		22/12/2022
R57	49	F	No/Yes	Neg	11/12/2021		22/12/2022
R58	63	F	No/No	Post	29/09/2021		22/12/2022
R59	52	M	No/No	Post	12/11/2021		14/11/2022
R60	48	M	No/No	Post	07/06/2021		11/08/2022
R61	55	M	No/Yes	Neg	02/03/2021		22/04/2022
R62	43	M	No/Yes	Neg	29/04/2020		17/05/2021

Long-COVID (LC) and Recovered (R). High blood pressure (HBP), Inflammatory bowel syndrome (IBD), Hospital ward (H. ward), Chronic obstructive pulmonary disease (COPD). Cytomegalovirus (CMV), neagtive (Neg), positive (post), male (M), and female (F).
